# Understanding Functional Redundancy and Promiscuity of Multidrug Transporters in *E. coli* under Lipophilic Cation Stress

**DOI:** 10.3390/membranes12121264

**Published:** 2022-12-14

**Authors:** Mohammad S. Radi, Lachlan J. Munro, Jesus E. Salcedo-Sora, Se Hyeuk Kim, Adam M. Feist, Douglas B. Kell

**Affiliations:** 1 Novo Nordisk Foundation Center for Biosustainability, Technical University of Denmark, Building 220, Kemitorvet, 2800 Kongens Lyngby, Denmark; 2 GeneMill, Shared Research Facilities, Faculty of Health and Life Sciences, University of Liverpool, Crown St., Liverpool L69 7ZB, UK; 3 Department of Bioengineering, University of California, 9500 Gilman Drive, La Jolla, San Diego, CA 92093, USA; 4 Department of Biochemistry and Systems Biology, Institute of Systems, Molecular and Integrative Biology, Faculty of Health and Life Sciences, University of Liverpool, Crown St., Liverpool L69 7ZB, UK

**Keywords:** adaptive laboratory evolution, *Escherichia coli*, multidrug transporters, lipophilic cations

## Abstract

Multidrug transporters (MDTs) are major contributors to microbial drug resistance and are further utilized for improving host phenotypes in biotechnological applications. Therefore, the identification of these MDTs and the understanding of their mechanisms of action in vivo are of great importance. However, their promiscuity and functional redundancy represent a major challenge towards their identification. Here, a multistep tolerance adaptive laboratory evolution (TALE) approach was leveraged to achieve this goal. Specifically, a wild-type *E. coli* K-12-MG1655 and its cognate knockout individual mutants Δ*emrE*, Δ*tolC*, and Δ*acrB* were evolved separately under increasing concentrations of two lipophilic cations, tetraphenylphosphonium (TPP^+^), and methyltriphenylphosphonium (MTPP^+^). The evolved strains showed a significant increase in MIC values of both cations and an apparent cross-cation resistance. Sequencing of all evolved mutants highlighted diverse mutational mechanisms that affect the activity of nine MDTs including *acrB*, *mdtK*, *mdfA*, *acrE*, *emrD*, *tolC*, *acrA*, *mdtL,* and *mdtP.* Besides regulatory mutations, several structural mutations were recognized in the proximal binding domain of *acrB* and the permeation pathways of both *mdtK* and *mdfA*. These details can aid in the rational design of MDT inhibitors to efficiently combat efflux-based drug resistance. Additionally, the TALE approach can be scaled to different microbes and molecules of medical and biotechnological relevance.

## 1. Introduction

Multidrug transporters (MDTs) provide microbes and other organisms the ability to defend themselves against external toxic compounds as well as toxic metabolites. A large arsenal of these MDTs is now known in prokaryotes, and mutations in these MDTs provide resistance to a wide range of xenobiotics including drugs [1,2]. Therefore, these transporters were considered as major contributors to microbial drug resistance, a huge worldwide health problem [3]. In addition, their efflux activities have been harnessed for improving product titers as well as enhancing host’s tolerance in biotechnological applications [4,5]. The significant role MDTs play in drug resistance relies on two main features: promiscuity, and functional redundancy. From their name, MDTs catalyze the efflux of structurally similar and dissimilar classes of drugs and other xenobiotics [6,7]. Additionally, activities of redundant MDTs are typically undetectable in wild-type microbes. However, they become important (i.e., observable) under increasing drug doses and/or after deletion of a potential transport system [8,9,10]. Thus, the functional redundancy of MDTs and their promiscuous nature can further serve as a starting point for evolving new functions, allowing for novel adaptative mechanisms. Although the advances in our genetic and structural understanding of different MDTs have been significant [6], revealing redundant MDTs and elucidating molecular details on multidrug recognition and transport in vivo remains challenging.

Tolerance Adaptive Laboratory Evolution (TALE) experiments have revealed precise information about the genetic basis underlying multiple phenotypes, including microbial drug resistance [11,12,13,14]. Previous TALE-based investigations showed how the rate and genotypic path to resistance varies across different controlled drug treatment regimens [14]. In conventional selection experiments, bacterial populations are subjected to fixed drug doses aiming at applying selective advantage for resistant mutants, but this typically identifies a single adaptive step [15,16,17,18]. However, after the generation of resistant mutants, higher drug doses are needed to maintain the selection pressure on the population’s current-higher resistance level and to reveal how multiple mutations can accumulate/enrich to confer stronger resistance. Obviously, the rate at which the inhibitory drug doses increase, which reflects the rate of evolution of resistance, can vary across the evolutionary trajectory and with the use of different drugs or strains with different starting genetic makeup [9,19,20,21]. Therefore, there is a need for a multistep long-term evolution experimental methodology in which the effective drug concentration is continuously tuned according to the actual rate of evolutionary adaptation. Technological innovations in liquid handling now facilitate rapid multistep TALE experiments, revealing long-term evolutionary paths of many organisms under various controlled lab conditions [11]. Additionally, the TALE approach has been introduced for the in vivo identification and modulation of membrane transporters, and the efficient generation of tolerance phenotypes of several microorganisms [22,23,24,25,26,27,28].

The knowledge base of multidrug transporter families in *E. coli* includes crystallographic data [6,29] of at least some of the major efflux pumps bound to drug substrate(s), which provide a foundation to explore similarities in drug recognition and drug export mechanisms. An example is *emrE* and the tripartite *acrAB/tolC* which are notable for their versatility in the efflux of several drugs in gram-negatives. Both efflux systems also share the binding and export of the two lipophilic cations tetraphenylphosphonium (TPP^+^) and methyltriphenylphosphonium (MTPP^+^) [30,31,32,33]. Thus, the promiscuous nature of the *emrE* and *acrAB/tolC* complex, and the two lipophilic cations TPP^+^ and MTPP^+^ are fundamental components in the experimental design used here. Accordingly, a wild-type *E. coli* K-12-MG1655 and its cognate knockout individual mutants Δ*emrE*, Δ*tolC,* and Δ*acrB* were evolved separately under increasing concentrations of TPP^+^ and MTPP^+^. The use of the wild type is hypothesized to signify the most reachable transport systems of both cations, while knockout strains would allow the discovery of redundant backup mechanisms that cover the loss of deleted transporters. The results showed that the multistep TALE approach allowed the generation of strains with high tolerance and that facilitated the discovery of several redundant MDTs and the mutational mechanisms underlying their modulation in vivo.

## 2. Materials and Methods

### 2.1. Strains, Chemicals, and Culture Media

The wild-type strain *E. coli* K-12 MG1655 (ATCC 47076) was initially spread on LB agar plates overnight, and unique single colonies were picked for further generation of single gene knock-out mutants based on using λ red recombineering and CRISPR-CAS9 as a counter selection. Adaptive laboratory evolution experiments were performed in M9 medium with 0.2% glucose, with added trace elements and vitamins, as described previously [22,27]. Tetraphenylphosphonium (TPP^+^) and methyltriphenylphosphonium bromide (MTPP^+^) were supplemented separately on an incremental basis. The manufacturer of the chemicals used was Sigma-Aldrich (St. Louis, MO, USA) unless otherwise stated.

To generate knockout strains, each chromosomal *emrE*, *tolC*, and *acrB* deletion was introduced using a temperature-sensitive pMP11 carrying λ-recombinases and *Streptococcus pyogenes* Cas9 [34,35]. Briefly, *E. coli* MG1655 was transformed with a temperature-sensitive pMP11. Lambda recombinases were induced with 0.2% arabinose at 37 °C for 45 min. Induced cells were transformed with a 200 ng gRNA plasmid for *emrE,* together with 100 pmol synthetic oligo, and each 200 ng FRT cassette containing each flanking 50 bp homologous sequence for *tolC* and *acrB*. Upon transformation, cells were recovered at 30 °C for 2 h. Cells were then transferred to 2 mL of LB containing ampicillin and chloramphenicol for the *emrE* knockout strain, and grown overnight at 30 °C. Each *tolC* and *acrB* knockout strain was selected on an LB agar plate containing kanamycin at 30 °C. *EmrE* knockout transformants were isolated by plating and validated by colony PCR and Sanger sequencing. The loss of guide RNA plasmid was achieved by growing confirmed isolates in LB containing ampicillin and 200 μg/L of anhydrotetracycline at 30 °C for at least 6 h. Loss of pMP11 was achieved by propagation at 37 °C. The loss of both plasmids was validated by checking the cell’s sensitivity to antibiotics. Plasmids and oligos used for strain reconstruction and PCR confirmation are listed in Supplementary Table S4.

### 2.2. Tolerance Adaptive Laboratory Evolution (TALE)

Laboratory evolution experiments were conducted on an automated platform using a liquid handling robot as previously described [22,26,27]. Briefly, four independent populations of *Ref*, *u*Δ*emrE*, *u*Δ*tolC,* and *u*Δ*acrB* were evolved separately on each of the cations. Cells were serially propagated (400 μL passage volume) in 15 mL (working volume) flasks of M9 minimal medium supplemented with 2 g/L glucose, kept at 37 °C through placement in a heat block and aerated by magnetic tumble stirrers at 1200 rpm. Cultures were monitored via changes in OD600 nm with a Tecan Sunrise plate reader (Tecan Inc., Männedorf, Switzerland) as a proxy for cell density. Growth rates were calculated for each flask using OD600 nm measurements and a best fit approximation for the growth using a three-phase model for lag, exponential growth, and stationary phase. The starting concentration of each amino acid was slightly above a predetermined minimum inhibitory concentration using the same liquid handler. There was a ‘resting phase’ parameter used to represent the minimum number of flasks at which the chemical concentration was not changed. This resting phase parameter varied between growing TALE replicates. Additionally, there was another parameter, ‘step increase’, which was set to define the increase in chemical concentration over the current amount once a culture met the increase criteria. When increased growth rate was achieved after a defined period of time (nearly equal to time it took for completion of two flasks, although this number varies according to the strain fitness) at a particular concentration, the cation concentration was increased when an observed fitness over a threshold was achieved (for example, a growth rate of 0.35 h^−1^). Occasionally, when there was not a considerable increase in the growth rate over the threshold, populations were left for the time it took for completion of three flasks. This process was repeated until a significant increase in tolerance was achieved. Each lineage was periodically validated by PCR, including the final flask, using the respective oligos (Supplementary Table S4).

### 2.3. Validation of Resistant Phenotypes and Cross-Cation Resistance

Evolved isolates were screened in high throughput for tolerance on selected concentrations of TPP^+^ and MTTP^+^ (Supplementary Table S1) using Growth Profiler (EnzyScreen BV, Leiden, The Netherlands). The sequenced clonal isolates derived from the endpoint populations (see Supplementary Table S2) were screened at a range of different concentrations (i.e., 8, 11, 14, 18, 24, 31, and 40 mM) of TPP^+^ and MTTP^+^. Colonies from the parental strains (i.e., *E. coli* K-12 MG1655 ‘*Ref*’ and its cognate knockout strains *u*Δ*emrE*, *u*Δ*tolC,* and *u*Δ*acrB* (denoted ‘unevolved’ knockout strains)), were used as a control. Cells were inoculated into 500 µL M9 glucose medium in deep well plates and incubated in a plate shaker at 37 °C and 300 rpm shaking. Later, cells were diluted 10× in M9 glucose medium, from which 30 µL was transferred to clear-bottom 96 half-deep well plates (EnzyScreen BV, Leiden, The Netherlands) containing M9 glucose medium with either of the two cations, such that the final concentration was equal to the range of the tested concentrations. For cross-cation resistance, cells were alternatively tested in the concentration gradient mentioned above in relation toeither cation toward which they were not evolved. The half-deep well plates were incubated at 37 °C with 225 rpm shaking in the Growth Profiler, with scans recorded at 20 min intervals. G-values were obtained from the plate images using the manufacturer’s software. G-values were converted to OD600 values using the formula

OD_600_ = a × (G_Value_ − G_Blank_)^b^

with the predetermined values a = 0.0024 and b = 1.69.

Growth rates were determined by using the R package Growthcurver [36]. Minimum inhibitory concentration (MIC) was determined as the lowest concentration at which less than 10% growth was detected relative to the growth in the basic M9 medium without the addition of either cation.

### 2.4. Whole Genome Sequencing and Mutational Analysis

To initiate the sequencing process, selected samples represent each evolved population at three time points of the evolutionary trajectory: the endpoint and two intermediate points approximately at the middle of the trajectory. Additionally, clonal isolates, derived from each selected population, was also selected. In total, 92 populations and 122 clonal isolates were re-sequenced. Selected cells were grown until reaching the stationary phase in M9 minimal medium supplemented with the highest concentration of the cation toward which the cells evolved. The flask numbers at which genomic DNA samples were taken for whole genome re-sequencing (WGS) are listed in Supplementary Table S2. Cell pellets obtained by centrifugation of 2 mL of culture and genomic DNA were extracted using PureLink^®^ Genomic DNA Kits (Invitrogen, Carlsbad, CA, USA) following the manufacturer’s protocol. The quality of extracted DNA was assessed with UV absorbance ratios using a nanodrop. The concentration of DNA was quantified using a Qubit ds-DNA high sensitivity assay. Paired-end resequencing libraries were generated using a 300 cycle (150 bp × 2) kit from Illumina (San Diego, CA, USA) with a loading concentration on a Nextseq 1.2 pico-Molar with 1% PhiX spike (Illumina, San Diego, CA, USA) of total input DNA. Raw sequence data reported in this paper have been deposited in the European Nucleotide Archive under Bioproject number PRJNA890058. Mutation identification was performed using a computational pipeline tool, as described in [37], based on *breseq* version 0.30.1 [38] in order to map sequenced reads to the reference strain (NCBI accession number NC_000913.3, K-12 MG1655). For the sequenced population samples, mutations were reported if they were over 25% frequency unless they were found in a clone isolated from a given population sample. This value was selected to focus on clearly causal mutations. Potential mutations were determined by comparing mutations from intermediate and endpoint samples, and identifying genes or intergenic regions that had multiple unique mutations (i.e., instances of parallel evolution) or were mutated across intermediate and endpoint isolates [37]. Mutations were filtered following a systematic logic (see Supplementary Figure S3) and categorized: (1) mutations targeting a unique ORF or ORF intergenic region identified for MDTs, (2) mutations affecting the upstream regulation of MDTs, and (3) other non-specific mutations which is important for the overall fitness (media adaptive mutations) or regulating osmotic stress. Amino acid substitutions were shown in the respective protein structures using PyMOL version 2.5.3 (https://pymol.org/2/ (accessed on 6 August 2022)).

### 2.5. Fluorophore Accumulation Transport Assay

Small flakes of bacteria in lysogenic broth (LB) glycerol stocks were spread on to LB agar plates containing antibiotic. Overnight single colonies were cultured in 5 mL of LB. The overnight cultures of bacteria were diluted 1:1000 in 5 mL of LB for slow growing strains (eTRef_A2F45I1, eTRef_A2F45I2, eMRef_A1F76I1, and eMRef_A1F76I2) or 1:2500 for the rest of the strains. After 2 h of incubation at 37 °C and shaking at 200 rpm, 50 μL of diluted cultures were transferred into 384-well plates already seeded with fluorophores at 2 μM final concentration (full list in Supplementary Note S1). After 15 min at 37 °C and shaking at approximately 1300 rpm, the 384-well plates were then sampled and analysed in the Intellicyt iQue (Sartorius, Göttingen, Germany) flow cytometer using all 13 channels. The flow cytometer protocol parameters were as follows: 2 min buffer (Qsol, Sartorius, Göttingen, Germany) priming with 10 s shaking at 2000 rpm, and 1 s sampling with two 0.5 s washes in buffer between each sample. Plates were shaken for 2 s every 12 wells while the probe sampled buffer. The final steps involved 30 s in flushing solution (Sartorius), 30 s in cleaning solution, and 60 s in deionised water. The prevalent cluster of light scattering events were gated in the forward scatter versus side scatter plot (population 1). Inside population one, a nested population was gated using the plot of forward scatter height versus forward scatter area and denoted population two. The gated population two would leave out most doublets. The median of the height distribution of the fluorescence signals (from all 13 channels) of population two for every sample were taken for data analyses, together with the number of counts. In total, 4 biological replicates were carried out for 17 bacteria strains with the reference strain ‘*Ref*’ present in every 384-well plate. Fluorescence signals were normalised against number of counts per sample. Data parsing, annotation, and visualisation were carried out using R.

## 3. Results and Discussion

### 3.1. The TALE Process

**Figure 1:**
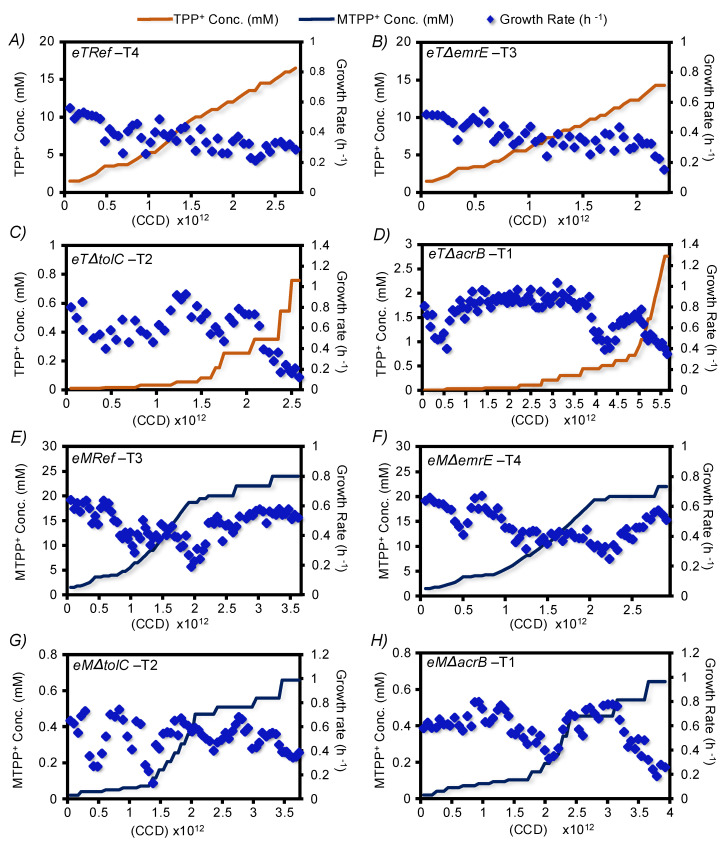
The full fitness trajectories of strains evolved independently under increasing concentrations of TPP^+^ and MTPP^+^. Depicted are the full fitness trajectories and the cation concentrations versus cumulative cell divisions (CCDs) experienced by a representative TALE lineage (abbreviated to T and the number indicate the replicate identifier) of each represented population. Each dot (bright blue diamonds) represents a calculated growth rate value of cells that were growing separately under increasing concentrations of TPP^+^ (orange lines, panel (**A**–**D**)), and MTPP^+^ (dark blue lines, panel (**E**–**H**)).

The TALE process was performed to generate strains that could tolerate elevated levels of two lipophilic cations, TPP^+^ and MTPP^+^, using a previously described methodology [22,26,27]. In brief, a wild-type *E. coli* K-12-MG1655 (denoted as ‘*Ref*’) was used as a parental strain to generate three mutant strains in which a promiscuous efflux pump was knocked out: *u*Δ*emrE*, *u*Δ*tolC,* and *u*Δ*acrB* (denoted ‘unevolved’ knockout strains). A total of 4 independent populations of each of *Ref*, *u*Δ*emrE*, *u*Δ*tolC,* and *u*Δ*acrB* were evolved separately on both cations for a total of 32 independent evolutions. TPP^+^-evolved strains were designated as follows: *eTRef*, *eT*Δ*emrE*, *eT*Δ*tolC,* and *eT*Δ*acrB* with ‘*T*’ denoting TPP^+^, and likewise, MTPP^+^ evolved strains will be designated as *eMRef*, *eM*Δ*emrE*, *eM*Δ*tolC,* and *eM*Δ*acrB* with ‘*M*’ denoting MTPP^+^. Cells were serially passaged during the late exponential growth under increasing concentrations of the cations until a significant increase in tolerance was achieved. The evolved strains displayed a divergence in the properties of the TALE experiments (Supplementary Table S1) in response to different treatment conditions. The observed fitness trajectories for representative TALE experiments for each strain, as well as the initial and final cation concentrations, are shown in Figure 1. Similar plots for the remaining lineages are shown in Supplementary Figures S1 and S2. Comparing the median values of the achieved final concentrations of both cations by all evolved lineages showed an enhanced tolerance compared to the ancestral strains. Specifically, the *eTRef* and *eT*Δ*emrE* populations showed an increase in TPP^+^ final concentrations equivalent to 9.2 and 7.5-fold, respectively, while both *eMRef* and *eM*Δ*emrE* populations showed a similar increase in MTPP^+^ concentration equivalent to 14-fold, relative to the starting concentration. These observations suggest that *eT*Δ*emrE* and *eM*Δ*emrE* evolved lineages exhibited a similar progression to the *eTRef* and *eMRef* with a marginal or no difference in tolerance towards both cations. Likewise, *eT*Δ*tolC* and *eT*Δ*acrB* populations showed an increase in TPP^+^ final concentrations equivalent to 256.8 and 1096.5-fold, respectively, while *eM*Δ*tolC* and *eM*Δ*acrB* populations showed an increase in MTPP^+^ concentration equivalent to 23- and 29-fold, respectively. However, this fold increase indicates an enhanced tolerance, the maximal cation concentrations achieved by these lineages were significantly lower (4.6- to 43-fold) compared to those achieved by *eT/MRef* and *eT/M*Δ*emrE*. In fact, the high sensitivity of the respective ancestral strains *u*Δ*tolC* and *u*Δ*acrB* at the onset of the TALE experiments towards either cation highlight the impactful disruption of *acrAB/tolC* tripartite, thus allowing selection of versatile backup transport mechanisms. On the real-time evolutionary trajectories, population fitness (i.e., calculated growth rate per each flask) fluctuated in response to the incremental dosage of either cation. However, the final growth rates of the endpoint populations ranged from 0.2 to 0.4 h^−1^. Consequently, the cumulative cell divisions (CCD) experienced by the evolved endpoint populations ranged between 2.5 × 10^12^ to 5.5 × 10^12^ regardless of the genotypic origin of the TALE lineages. The use of CCD has previously been shown as a more meaningful scale for the time coordinate of a TALE than generations [39], as mutations occur predominantly due to DNA polymerase errors in genomic replication during cell division [40]. Screening of the evolved populations was subsequently performed to validate the overall tolerance compared to the ancestral strains, and to demonstrate possible cross-cation resistance.

### 3.2. Validation of Resistant Phenotypes and Cross-Cation Resistance

**Figure 2:**
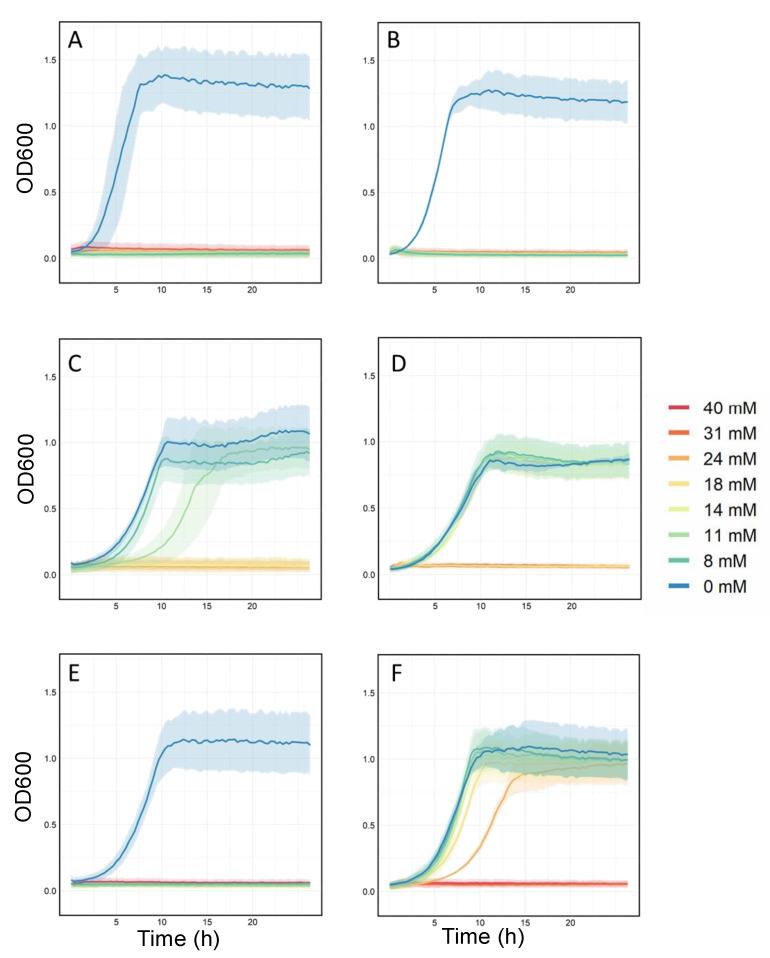
Phenotypic characterization of the evolved strains. Depicted is the growth curves of representative clones derived from *eTRef* and *eMRef* populations in comparison to their ancestral strain *Ref*. The phenotypic assessment was performed in the indicated concentration gradient of TPP^+^ and MTPP^+^. (**A**) Growth of ‘*Ref*’strain in TPP^+^. (**B**) Growth of ‘*Ref*’ strain in MTPP^+^. (**C**) Growth of *eTRef* evolved strain in TPP^+^. (**D**) Growth of *eTRef* evolved strain in MTPP^+^. (**E**) Growth of *eMRef* evolved strain in TPP^+^. (**F**) Growth of *eMRef* evolved strain in MTPP^+^. Shaded area represents standard deviation (*n* = 4).

**Figure 3:**
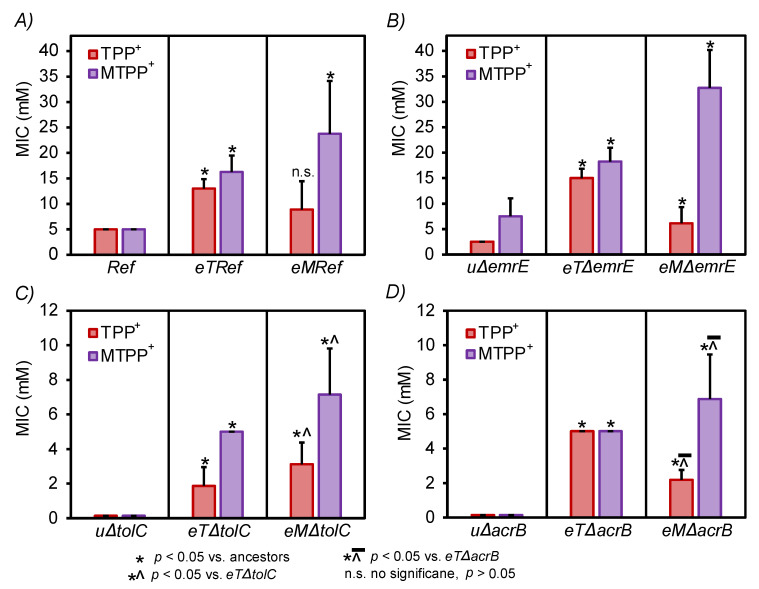
Validation of tolerance phenotypes and cross-cation resistance of the evolved strains. Depicted are the average MIC values (mM) of both TPP^+^ (red bars) and MTPP^+^ (violet bars) experienced by the evolved strains versus their respective ancestors. Strains were tested in the respective cation toward which they were evolved (to confirm tolerance phenotype) and tested separately on the alternative cation toward which they weren’t evolved (to test cross-cation resistance). (**A**) MICs of *eTRef* and *eMRef* compared to their ancestor, ‘*Ref*’. (**B**) MICs of *eT*Δ*emrE* and *eM*Δ*emrE* compared to their ancestor, *u*Δ*emrE*. (**C**) MICs of *eT*Δ*tolC* and *eM*Δ*tolC* compared to their ancestor, *u*Δ*tolC*. (**D**) MICs of *eT*Δ*acrB* and *eM*Δ*acrB* compared to their ancestor, *u*Δ*acrB.* MIC was determined as the lowest concentration at which less than 10% growth was detected relative to the growth in the basic M9 medium without the addition of either cation. Error bars represents standard deviation (*n* = 4). Symbols above the bars indicate statistcal significance between groups (*): *p* < 0.05 vs. ancestors, (^): *p* < 0.05 vs. *eT*Δ*tolC,* (⊼): *p* < 0.05 vs. *eT*Δ*acrB*, and (n.s.): no statistical significance *p* > 0.05 (Mann-Whitney U test).

The enhanced tolerance phenotypes of the evolved lineages were further validated and compared to their respective ancestors. Additionally, a demonstration of possibly acquired cross-cation resistance was performed by testing the growth of the evolved cells on the alternative cation toward which these cells were not explicitly evolved. For these experiments, two randomly selected clones derived from each TALE lineage endpoint population (64 clones in total) were phenotypically screened in a broad range of TPP^+^ and MTPP^+^ concentrations (i.e., 0, 8, 11, 14, 18, 24, 31, and 40 mM). An example of the phenotypic screen of *eTRef* and *eMRef*, in comparison to their respective ancestor, is shown in Figure 2. The MIC was used as a metric for the assessment of both cation tolerance and acquired cross-cation resistance. The determined average MIC values of both TPP^+^ and MTPP^+^ towards all evolved clones are summarized and compared to controls as shown in Figure 3. Generally, both TPP^+^ and MTPP^+^ evolved clones which showed a significant tolerance compared to their ancestors, and developed an acquired cross-resistance towards the alternative cations. Specifically, *eTRef* and *eMRef* showed a 1.6- and 3.75-fold increase in MIC of TPP^+^ and MTPP^+^, respectively, relative to their ancestors. The screened clones derived from one of the *eMRef* lineages (i.e., T1) showed an exception with an MIC value equivalent to 40 mM of MTPP^+^ which is 68% above the mean (but consistent), and even 80% higher than the maximum MTTP^+^ concentration achieved during the respective TALE experiment. Similarly, *eT*Δ*emrE* and *eM*Δ*emrE* showed an increase in MIC values of TPP^+^ and MTPP^+^ with 5- and 3.37-fold, respectively, compared to their ancestors. Despite the observed susceptibility to both cations in the duration of the TALE experiments, the reported MIC values of TPP^+^ for *eT*Δ*tolC* and *eT*Δ*acrB* showed 12.4 and 34.7-fold increase, respectively, while MIC values of MTPP^+^ for *eM*Δ*tolC* and *eM*Δ*acrB* showed 50 and 48-fold increase, respectively, all compared to their ancestors.

Cross-cation resistance was apparent among the majority of the evolved isolates with different degrees (Figure 2). The MIC values generated for clones derived from three TPP^+^ evolved lineages (i.e., *eTRef*, *eT*Δ*emrE*, and *eT*Δ*acrB*) suggested a high degree of acquired cross resistance against MTPP^+^ (with similar or marginally different MICs). Exceptionally, *eT*Δ*tolC* isolates retained 1.6-fold increase in MIC values of MTPP^+^ (a cation toward which these clones were not evolved) compared to MIC values of TPP^+^. Clearly the reported MIC values of TPP^+^ were lower in all tested clones compared to MTPP^+^, and consequently all TPP^+^ clones retained high MICs of MTPP^+^ in cross tolerance trials. These results are consistent with the fact that the level of cross resistance depends on the degree of susceptibility of the host bacterium and possibly the underlying resistance mechanism has no absolute value [41]. However, a reversed pattern of cross resistance has been shown in all MTPP^+^ evolved mutants (i.e., *eMRef*, *eM*Δ*emrE*, *eM*Δ*tolC,* and *eM*Δ*acrB*). The higher MIC values of MTPP^+^ experienced by these clones accompanied by lower MIC values of TPP^+^ in all cross-resistance trials (although generally retained higher MICs compared to their respective ancestors). The genetic basis underlying the overall enhanced tolerance phenotypes was determined by whole genome resequencing.

### 3.3. Whole Genome Re-Sequencing and Mutational Analysis

**Figure 4:**
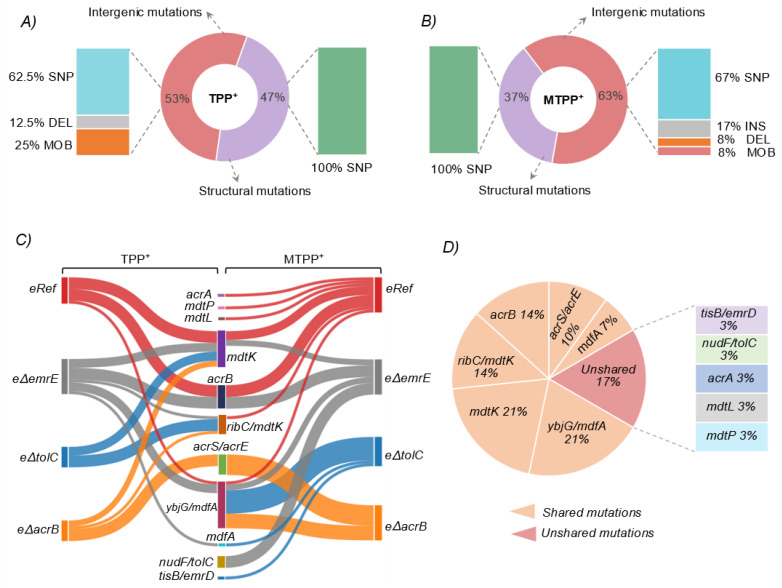
Overview of identified mutations affecting MDTs. (**A**) Info chart of mutations targeted MDTs in TPP^+^ evolution experiments. Mutations split into 47% of structural (coding) mutations and 53% of intergenic (non-coding) mutations. All structural mutations were SNPs, while intergenic mutations categorized as 63% SNPs, 13% DELs, and 25% MOB. (**B**) Info chart of mutations targeted MDTs in MTPP^+^ evolution experiments. Intergenic mutations represented 63%, and varied between SNPs (67%), INSs (17%), DELs (8%), and MOB (8%). The remaining share (37%) represented the structural mutations, and all were SNPs. (**C**) A Sankey diagram linking the mutations occurred in all strains tested in TPP^+^ and MTPP^+^. Line width represent the number of unique occurrences in which a given ORF or ORF intergenic region was mutated. Genes or genetic region that lack a dual link between the two conditions are unshared. (**D**) Info chart illustrating the mutated ORF or ORF intergenic regions that are shared between the strains tested in both conditions (orange pies), and the unshared mutations (red pie), and the bars enumerate the unshared genes/genes intergenic regions. Percentages are rounded to the whole number. (Abbreviations: SNP: single-base substitution, MOB: mobile element insertion, DLE: deletion, and INS: insertion).

Whole genome resequencing (WGS) was used to determine the genetic basis underlying the increased resistance phenotypes, focusing on mutations related to MDTs. Potential mutations were determined by comparing mutations from intermediate and endpoint samples. and identifying genes or intergenic regions that had multiple unique mutations (i.e., instances of parallel evolution) or were mutated across intermediate and endpoint isolates of the same lineage [37]. Different time points (including all screened clones) are listed in Supplementary Table S3. Mutations were filtered following a systematic logic (see Methods and Supplementary Figure S3) and categorized. (1) Mutations targeting a unique ORF or ORF intergenic region identified for MDTs (Table 1 and Figure 4), (2) mutations affecting the upstream regulation of MDTs (Supplementary Table S3), and (3) other non-specific mutations which were important for the overall fitness (media adaptive mutations) or regulating osmotic stress (Supplementary Data S1).

The first category highlighted nine distinct MDTs throughout the evolution experiments, including *mdtK*, *mdfA*, *acrB*, *acrE*, *tolC*, *acrA*, *emrD*, *mdtL*, and *mdtP*, ranked from highest to lowest number of occurrences. As visualized in (Figure 4A), TPP^+^ evolved mutants showed a diverse number of mutations that split into 47% of structural (coding) mutations and 53% of intergenic (non-coding) mutations. Further, all structural mutations were single nucleotide polymorphisms (SNPs), while intergenic mutations diversified into 63% SNPs, 13% small deletions (DEL), and 25% mobile element insertion (MOB). Likewise, intergenic mutations observed in MTPP^+^ evolved mutants were prevalent (63%), as shown in (Figure 4B), and these mutations varied between SNPs (67%), insertions or INS (17%), DEL (8%), and MOB (8%). The remaining share (37%) represented the structural mutations, and all were SNPs. A detailed enumeration of these mutations and their possible effects on MDTs will be further presented in Section 3.3.1 and Section 3.3.2. There was a major overlap (i.e., similarity) in both mutation types and the affected MDTs (either ORF or ORF intergenic region) among different strains evolved in each condition (Figure 4C,D). There was approximately 87% of mutations that commonly shared between different strains evolved in both conditions. This initially hinted at the overlapping specificity/multiplicity of the reported MDTs and explained the observed cross-cation resistance (Section 3.2).

The second category highlighted two frequently mutated transcriptional repressors *acrR* and *marR* that were targeted by mutations in their coding region. Mutations in *acrR* occurred frequently in *eTRef*, *eT*Δ*emrE*, *eMRef,* and *eM*Δ*emrE* evolved mutants, while mutations in *marR* occurred only in *eM*Δ*emrE* evolved mutants. Mutations targeted *acrR* were diverse including SNPs, DELs, small insertions—INS, and mobile element insertions—MOB). Examining the impact of two SNPs (GAA→TAA and TCG→TAG) and a MOB mutation suggested a loss of *acrR* function due to the generation of premature stop codons or disruption of the *acrR* ORF by an IS*2* element insertion. The impact of other mutations was assessed computationally, as previously described [42], and these mutations were predicted to either target a highly conserved region or were destabilizing amino acid substitutions. Likewise, mutations in the coding region of *marR* (SNPs resulted in amino acid substitution) either affected the conserved region of the protein or *marR* dimer interaction interface. There were two instances of *eM*Δ*emrE* parallel evolutions where mutations in both *marR* and *acrR* co-occurred. It has been reported that mutation in their encoding genes, *marR* and *acrR*, can maintain the overactivity of the *AcrAB-TolC* pump via overexpression of the *acrAB* operon [43,44]. Therefore, the partial or whole inactivation of both *acrR* and *marR* reported here suggests a possible mitigation of transcriptional repression of *acrAB.* It is also worthy to mention that mutations in *acrR* and *marR* co-occurred with structural mutations in *acrB* ORF (but not in the *acrB* ORF intergenic region) in the same evolved mutants (i.e., *eTRef*, *eT*Δ*emrE*, *eMRef,* and *eM*Δ*emrE*).

The third category represented non-specific mutations that are not directly associated with multidrug transport (Supplementary Data S1). The ubiquitously occurring mutations of this category are generally important for enhancing fitness and cell survival under treatment with inhibitory chemicals and molecular regulation of stress response in *E. coli* [45,46,47,48,49,50]. Consequently, these mutations were not considered here due to the lack of potential relationship with multidrug transport.

There were four hypermutator samples derived from flask 59 and 67 of *eMRef* replicate T1. These isolates had 433 mutations as compared to the average mutations 15 ± 4 for the non-hypermutating samples with standard deviation shown between replicates. However, mutations in MDTs found in these hypermutators were considered for further discussion (Section 3.3.1 and Section 3.3.2) in case they added to the overall mutations directly associated with MDTs. This particular genotype might explain the exceptional MIC values reported for these mutants during tolerance validation experiments (Section 3.2).

#### 3.3.1. Structural Mutations Affecting MDTs

Structural mutations in MDTs represented a sizable proportion of mutations reported in TPP^+^ and MTPP^+^ evolutions, with 47% and 37% of total mutations, respectively (Figure 4A,B). The ORF of a total of nine distinct MDTs was targeted by structural mutations, most of which were conservative missense mutations (Table 2). These MDTs included *acrB*, *mdtK*, *mdfA*, *acrA*, *mdtL*, and *mdtP* and ranked from most frequently mutated to least frequently mutated based on their occurrence among all trials.

**Figure 5:**
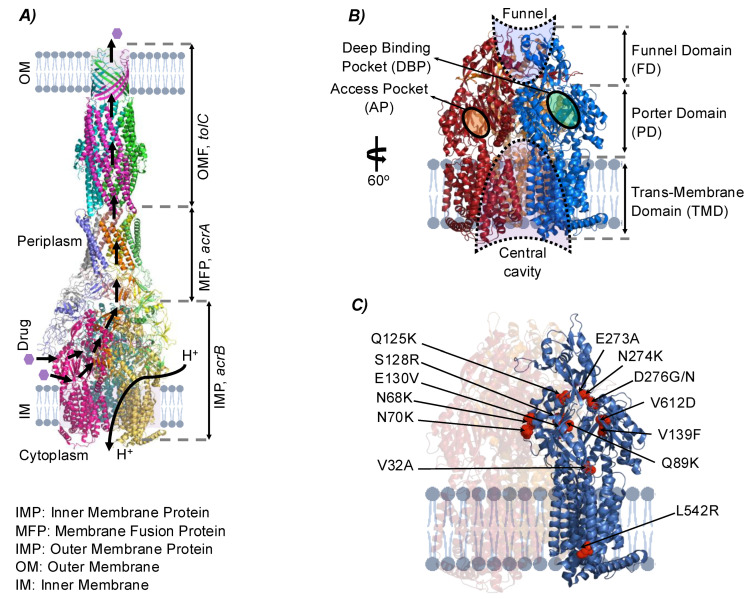
Overview of identified mutations affecting *acrB*. (**A**) The *acrAB/tolC* multidrug efflux pump complex (PDB: 5v5s). The complex consists of a resistance-nodulation-division (RND) family inner membrane transporter *acrB*, a membrane fusion protein (MFP) *acrA,* and a multifunctional outer membrane channel *tolC*. The complex is a major contributor to intrinsic resistance for many compounds (antibiotics, antiseptics, detergents, dyes, and others) and the transport process is proton dependent. (**B**) The architecture of *acrB* inner membrane protein (PDB: 4DX5). Depicted is the *acrB* homotrimer showing the three main domains, namely, a funnel (FD), a porter (PD), and a transmembrane domain (TMD). The PD of each monomer has two binding pockets, access pocket (AP) and deep binding pocket (DBP). Generally, the transport cycle of a molecule starts with binding to the AP, tunneling to the DBP, and extrusion through a funnel like structure toward *tolC* via *acrA.* This transport cycle is aided with a synchronous confirmational changes in the TMD due to a proton release to the cytoplasm. Some compounds might also enter the central cavity via the vestibules between the protomer interfaces at the level of the membrane plane. (**C**) Mutational changes in the *acrB.* Highlighted is a monomer (blue) with all amino acid substitutions (red) reported in *eMRef* and *eM*Δ*emrE* evolved mutants. All mutations (replicates represented once) were located in the PD and possibly affect the DBP except only one mutation that was found in the TMD. Panels (**A**,**B**) are adapted from [59].

The RND (resistance-nodulation-division) family protein, *acrB*, is the inner membrane component of the *acrAB*/*tolC* tripartite, proton dependent, drug efflux pump [52]. The crystal structure of *acrB* was determined [53,54,55] with bound ligands rhodamine 6G, ethidium, dequalinium, and ciprofloxacin [56,57] and with the secondary bile acid, deoxycholate [58]. As shown in Figure 5, *acrB* is structurally divided into a funnel domain (FD), important for *acrB* trimerization and a trans-membrane domain (TMD) responsible for the energy transduction to facilitate drug transport. A further division is the porter domain (PD) that contains the binding pockets (access pockets (APs) and deep binding pockets (DBPs)) and mediates substrate uptake, recognition, and translocation [59]. All mutations that targeted *acrB* were located the PD (13 amino acid substitutions excluding replicates), except one mutation that was found in the TMD (Figure 4C). Most of the mutations that targeted the PD affect specifically the DBP of *acrB.* Consistent with previous studies [60,61,62,63], the specific amino acid residues E130, Q89, V139, N274, D276, and V612 were essential for interaction with several antibiotics and inhibitors. In addition, the amino acid substitution V139F, which is shared between *eMRef* and *eM*Δ*emrE* evolved mutants, was found in isolates of *E. coli* BW25113 and subjected to in-lab evolution against chloramphenicol [9]. Interrogating the observed amino acid substitutions generally indicate a drastic shift in hydropathy indices (either from low to high or vice versa in different locations near DBP), a minor preference to polar amino acids, and a moderate shift toward acidic amino acids (Table 2). This trend is in line with the nature of the interaction between the known ligands and the DBP of *acrB* [59]. The DBP is predominantly lined by weakly hydrophobic and weakly polar residues that are predicted to create a versatile and adaptable environment with numerous so-called multifunctional sites [59,64,65]. Accordingly, the amino acid alterations reported here are suggested to provide either complementarity towards parts of the cations’ structure, or flexibility in interaction to accommodate and facilitate their efflux.

**Figure 6:**
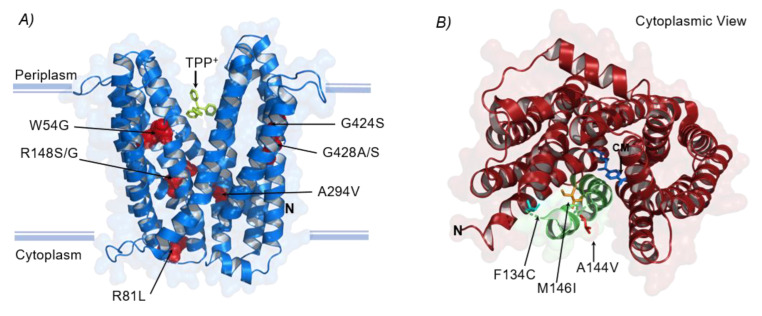
Overview of identified mutations affecting *mdtK* and *mdfA*. (**A**) Side on view of *mdtK* with TPP^+^ (lemon-green sticks) bound in the periplasmic side. Depicted is the predicted architecture of *mdtK* aligned with the crystal structure of the homologous multidrug effluxer *norM* from *Neisseria gonorrhoeae* in complex with TPP^+^ (PDB 4huK). Amino acid substitutions (red spheres) represent all mutations reported (replicates represented once and details are in Table 2). (**B**) Cytoplasmic view of *mdfA* in the outward conformation (PDB 4zow) in complex with CM (blue sticks). Green ribbon represents TM5, and arrows indicate the observed amino acid substitutions.

The MATE (Multidrug And Toxic Compound Extrusion) family protein *mdtK* is an inner membrane, proton (or sodium) dependent, drug efflux pump within the Multidrug/Oligosaccharidyl-lipid/Polysaccharide (MOP) flippase superfamily [52,66]. Structural mutations in *mdtK* were shared among many strains and conditions including *eTRef*, *eMRef*, *eT*Δ*emrE*, *eM*Δ*emrE*, *eT*Δ*tolC*, and *eM*Δ*acrB* evolutions (Table 2 and Figure 4). In total, six distinct amino acid substitutions were reported with prevalence given to residue R148 that was frequently mutated to glycine and uncommonly to serine. Both alterations indicate a shift in the hydropathy index from −4.5 (for arginine) to −0.4 and −0.8 for glycine and serine, respectively. Additionally, both alterations alleviated the positive charge conferred by the strongly basic arginine residue. Substitutions of arginine in other locations were reported (e.g., R81C and R81L) which showed a similar change in properties as the previous ones. Other substitutions include G428A/S which was found only in *eT*Δ*tolC* and *eM*Δ*acrB* mutants, respectively, and three different substitutions (i.e., W54G, A294V, and G224S) that were found only in *eM*Δ*acrB* evolved mutants. It is not immediately clear how these mutations affect the transport process due to the lack of information about *mdtK* structure. However, a homolog called *norM* found in *Neisseria gonorrhoeae* was crystalized in complexes with different cations including TPP^+^ [67,68]. The structure of *mdtK* was predicted [69,70] and aligned with *norm,* as shown in Figure 6A. Both substitutions R148G/S (in the central cavity) and R81L (facing the cytoplasmic side) are likely located in the substrate permeation pathway. The removal of the positive charge conferred by arginine suggests an alleviation of unfavorable interaction during the export of positively charged cations. In general, none of the newly substituted amino acids are aromatic, specifically all new variants around the multidrug binding cavity. This is consistent with the nature of the multidrug binding cavity of *norM*, as it was found to be festooned with four negatively charged amino acids, unlike other multidrug effluxes that utilizes aromatic amino acids for multidrug recognition [67].

The MFS (Major Facilitator Superfamily) family protein *mdfA* is an inner membrane protein that transports several drugs in exchange for protons and other cations [71,72]. The recently reported structure of chloramphenicol (CM)-bound *mdfA* shows that the transmembrane helices allow alternating access to the cytoplasmic and the periplasmic sides following a rocker-switch mechanism [73,74]. The transport of substrates across these helices mainly follows a transition between outward open (O_o_) and inward facing (I_f_) states. Three amino acid substitutions were observed in *mdfA* including A144V, F134C, and M146I that occurred with equal frequency in samples derived from two evolved mutants *eT*Δ*emrE* and *eM*Δ*tolC* (Table 2 and Figure 6B). Interestingly, all these residues are in the *mdfA* transmembrane helix (TM5) whose conformation significantly differs between the two states O_o_ and I_f_. With a special reference to residue M146, which was found to be responsible for the largest deviation in TM5 during the transport cycling [73]. Further, the hydrophobic side chain of residue M146 (TM5) was crucial for local twisting and rearrangement of the hydrophobic core of N-terminal domain of *mdfA* by resting against the phenolic side chain of Y127 (TM4) during drug (CM) transport. The M146I mutation reported here indicates a drastic change only in hydropathy (i.e., from 1.9 to 4.5), with roughly equal polarity and charge, and this may be delineated as an enhancement in *mdfA* activity based on the mechanism explained earlier. Even though CM transport by *mdfA* is electrogenic, while the transport of TPP^+^ is electroneutral, none of the protonation adjusting residues were mutated indicating that these mutations particularly modulate cation binding and efflux. It is worth mentioning that the mutations M146I and A144V co-existed with most mutations affecting the ORF intergenic region of *mdfA* in samples derived from *eM*Δ*tolC* and *eT*Δ*emrE*, respectively (see Figure 4C), indicating the high selection pressure due to the lack of the respective drug efflux components.

Three rarely mutated MDTs were reported: (1) *acrA*, the periplasmic lipoprotein component of the *acrAB/TolC* and *acrAD/TolC* multidrug efflux pumps in *E. coli*, (2) *mdtL*, an inner membrane multidrug efflux pump that belongs to the MFS, and (3) *mdtP*, a putative multidrug efflux outer membrane channel [29]. Despite the drug efflux activities of these transporters, their occurrence was rare and was reported in only a few samples of *eMRef* evolved mutants. This may either reflect their minor contributions to the overall resistance phenotype, or their eradication (wash out) due to enrichment of more efficient modulating mutations affecting other drug effluxes, as described earlier (see also Section 3.3.2). The second possibility is more plausible as these mutations were found only in the sequenced endpoints, and because the highest concentrations of MTPP^+^ were reported in the *eMRef* lineages (as described in Table S1). This assumption might be explained in light of a general recruitment of such MDTs to collectively alleviate high MTPP^+^ external supplementation.

#### 3.3.2. Intergenic Mutations Affecting MDTs

The overall mutations occurred in the intergenic region *ybjG*/mdfA, *ribC*/*mdtK*, and *acrS*/*acrE* (see Table 1 and Figure 4) and suggest a strong selective pressure and evidence of parallel evolution at these loci. Two different mutational types were captured in the intergenic region *ybjG*/*mdfA*, which included three SNPs and one mobile element insertion (MOB) (Table 1). Two SNPs were similar (i.e., C > A), but occurred at two different locations, namely 30 and 12 bps upstream of *mdfA*. The first SNP occurred in samples derived from *eTRef* evolved mutants, while the later SNP occurred in samples derived from both *eTRef* and *eT*Δ*emrE.* Unexpectedly, the effect of these two SNPs on the promotor region of *mdfA* is not clear despite the closeness of these SNPs to the proposed −10 consensus region of *mdfA* promotor [75]. However, the later SNP (i.e., −12) clearly affects a putative ribosome-binding site (RBS), GGCG, which is located 14 to 11 bps upstream of *mdfA* start codon [75]. This RBS is a suboptimal ShineDalgarno sequence [76], but the −12 SNP changed its sequence to GGAG which was found to stimulate the translation of some genes by several folds [77,78]. This is likely suggestive of a similar effect resulting in *mdfA* overexpression, which is consistent with the experimental context presented here. The remaining SNP (T > G) was 57 bps upstream of *mdfA* and found in only one sample of the evolved mutant *eMRef*. The effect of this SNP is unclear as it did not target any essential regions that might affect either *mdfA* or *ybjG*. The remaining MOB mutation was associated with the mobile genetic element IS*30* and was reported in two parallel replicates of *eT*Δ*emrE*. The IS*30* insertion occurred 119 bps upstream of *mdfA*. A similar instance was reported when *E. coli* MG1655 was exposed to long-term low-shear modeled microgravity and background chloramphenicol exposure, but IS*30* insertion occurred −118 bp upstream of *mdfA* [79]. The authors interpreted this mutation as affecting the transcription of the downstream gene, *ybjG* (undecaprenyl pyrophosphate phosphatase), but this interpretation was not in line with their experimental context. It has been proven experimentally that IS*30* insertions were found to generate promoter sequences at the junctions of the IS elements with the target sequences [80,81,82]. There is a −35 control signal of a potential promoter sequence contained within the left-hand terminal inverted repeat of IS*30*, while the −10 region is contained in an internal symmetrical sequence [83]. The IS*30* insertion site reported here is 14 bp upstream of cmrp2, a proposed −10 consensus of a promoter controlling the expression of *mdfA* [84]. This rearrangement implies that IS*30* insertion would enhance *mdfA* expression, which is a more plausible assumption that is consistent with the experimental conditions used here. However, this requires further study.

In total, two different mutational types were manifested in the intergenic region *ribC*/*mdtK*, which included four SNPs and a small DEL (Table 1). Three SNPs were similar (i.e., G > C) and occurred in the same intergenic site, specifically 111 bps upstream of *mdtK*. This SNP was commonly shared between samples derived from *eT*Δ*emrE*, *eT*Δ*tolC,* and *eMRef* evolved mutants, while the remaining SNP (i.e., A > C) was 25 bps upstream of *mdtK* and occurred only in samples derived from *eT*Δ*acrB* evolved mutants. The additional small DEL was 15 bps in length and occurred within the intergenic region 130 and 71 bps upstream of *ribC* and *mdtK*, respectively. Interestingly, the prevalent SNP reported here (i.e., G > C) and the small DEL co-occurred in four samples (out of six) derived from *eT*Δ*tolC* evolved mutants. Such allelic heterogeneity may infer a high selection pressure at these loci and the observed resistance of these mutants might be enhanced when both mutations co-exist. The effect of all these mutations was examined based on previous reports. There are two computationally identified promoters for *mdtK*, mdtkp9, and mdtkp10 [85]. The 3 prevalent SNPs reported here are all located only 4 bases downstream of the −35 signal of mdtkp9 and the 15 bp DEL is located 3 bp downstream of the −10 signal of the same promoter. Due to the lack of supportive experimental evidence of the precise identities of both mdtkp9 and mdtkp10, it was difficult to infer the possible effect of the reported mutations on either flanking genes. However, intergenic regions are home to many functional elements required for resistance determinants in vivo and are maintained by purifying selection in many bacterial species [86,87,88]. Additionally, increased expression of *mdtK* in an *E. coli* strain lacking *acrAB* resulted in a 32-fold increase in resistance to TPP^+^ [89] and other antibiotics and dyes [90,91,92] which are similar and relevant experimental conditions. Thus, the reported mutations are expected to affect the expression of the multidrug effluxer *mdtK* rather than riboflavin synthase *ribC*.

The Intergenic region *acrS/acrE* was targeted by two different mutation types (i.e., MOB and SNP) which occurred only in *eT*Δ*acrB* and *eM*Δ*acrB* evolved mutants. The MOB mutations were more prevalent and associated with three different mobile genetic elements IS*1*, IS*2,* and IS*5*. Specifically, the IS*1* insertion occurred 138 bps upstream of the *acrE* initiation codon and found in samples derived from *eT*Δ*acrB* mutants only. The IS*2* insertion occurred 86 or 90 bps upstream of the *acrE* initiation codon, accompanied by doubling of the pentamer GTAGG, and this mutation reported in both *eT*Δ*acrB* and *eM*Δ*acrB* evolved mutants. In a particular instance, an IS*2* insertion occurred 10 bps upstream of *acrF*, a multidrug efflux pump located 11 bps upstream of *acrE* and belonged to the same operon (*acrEF*). This mutation occurred in all intermediate and endpoint samples (i.e., fixed mutation) of only one TALE replicate of *eT*Δ*acrB* evolution. The IS*5* insertion occurred twice: 163 and 94 bps upstream of the initiation codon of *acrE*. The latter mutation was found in samples derived from *eM*Δ*acrB* mutants only. In addition, there were two different SNPs, T > G and G > A that occurred 92 and 86 bps upstream of the *acrE* start codon. The later SNP (i.e., G > A) co-occurred with the IS*5* insertion mutation in five samples derived from three parallel TALE replicates of *eM*Δ*acrB*, while both SNPs co-occurred with the IS*5* insertion mutation in two samples derived from two parallel TALEs of the same strain *eM*Δ*acrB*. Both SNPs are likely to affect the binding of the transcriptional binding regulator Nac, that might inhibit the transcription of *acrE*. The effluxer *acrE* shares 65% amino acid identity with the membrane fusion protein *acrA* of the *acrAB* efflux pump complex, while the adjacent gene *acrF* shares 77% amino acid sequence identity with *acrB* of the *acrAB* system [93,94]. Both *acrEF* are not expressed at significant levels in wild-type *E. coli* K12 [95,96], although there is strong evidence that *acrEF* is expressed at a higher level upon integration of IS*1* and IS*2* into the upstream region of the operon [97]. In the later publication, the authors found IS*1* and IS*2* insertions at similar (−90) and closer (−187) sites, both relative to *acrE*, which is consistent with the results reported here. The presence of potential promoters and ribosomal binding sites within the IS*1* and IS*2* elements was proven experimentally as the main reason for increased expression of *acrEF* [97,98]. In a similar instance, in vivo development of tigecycline resistance emerged in *Klebsiella pneumoniae* clinical isolates upon integration of the IS5 upstream of a multidrug effluxer, *kpgABC* [99]. Moreover, an episomal expression of *acrEF* in strains lacking *acrB* resulted in an increased resistance to a wide range of antibiotics [92,100,101]. The mutational events occurred here, and the structural similarity of both *acrEF* with *acrAB* and the sole occurrence of these mutations in *acrB* null mutants, hint at the conservation of the *acrEF* operon as a backup strategy that compensate the loss of *acrAB*.

Two unshared mutations in the intergenic regions *nudF/tolC* and *tisB/emrD* were found in *eM*Δ*emrE* and *eM*Δ*tolC* TALE experiments, respectively (Figure 4C,D). First, the mutations found in the intergenic region *nudF/tolC* included one prevalent DEL (i.e., 1 bp deletion), and a less frequent (but fixed) SNP (G > A) 180 and 141 bps upstream of *tolC*, respectively. The effect of the DEL mutation is not clear as it did not target any known essential controlling elements for the transcription of either *tolC* or *nudF*. However, the G > A SNP altered the first nucleotide of the DNA-binding site for the transcriptional dual regulator PhoP that activated *tolC* transcription [84]. The increased demand of *tolC* as a channelling protein under stress conditions may justify a possible increase in *tolC* expression due to this mutation. Despite the activation of *acrB*, *mdtK*, and *mdfA* in *eM*Δ*emrE* evolutions, *acrB* is the only *tolC*-dependant effluxer in this list and its activation was based on structural mutations in the distal binding pocket and a possible overexpression due to inactivation of *acrR*. In addition, the co-occurrence of such an SNP with any of the reported *acrB* variants was rare (i.e., found in one sample out of seven). This observation would indicate that the enhanced activity of *acrB* is not concurrently associated with an increased expression of *tolC*, or a *tolC*–independent efflux activity of *acrB* as previously hinted [102]. Second, there was only one rare SNP (C > A), 58 bps upstream of *emrD*, in a well-known inner membrane multidrug effluxer [71,103,104]. However, this SNP is 18 bps downstream of the absolute position of a putative transcription start site [105] and it does not seem to affect any known essential transcription controlling elements of *emrD*.

### 3.4. Estimating Changes in Membrane Transport in Terms of Mutational Convergence

The changes in membrane transport based on the acquired converged mutations were evaluated using a flow cytometry-based phenotyping assay, as previously described [106,107]. Briefly, a set of 46 fluorophores known to accumulate intracellularly in *E. coli* were chosen to estimate the effect of the converged mutations associated with MDTs in both TPP^+^ and MTPP^+^-evolved mutants. To limit the scale of the experiment, 16 endpoint clones derived from *eTRef* and *eMRef* evolved lineages were used. These clones were recognized to possess most of the converged mutations associated with MDTs and their regulation (see Section 2.3). Fluorophore signal ratios were used as a metric to contrast the intracellular accumulation of fluorophores in the evolved mutants versus their ancestral strain ‘*Ref*’. Fluorescence signal ratios below zero indicate lower intracellular accumulation of fluorophores, and *vice versa*.

**Figure 7:**
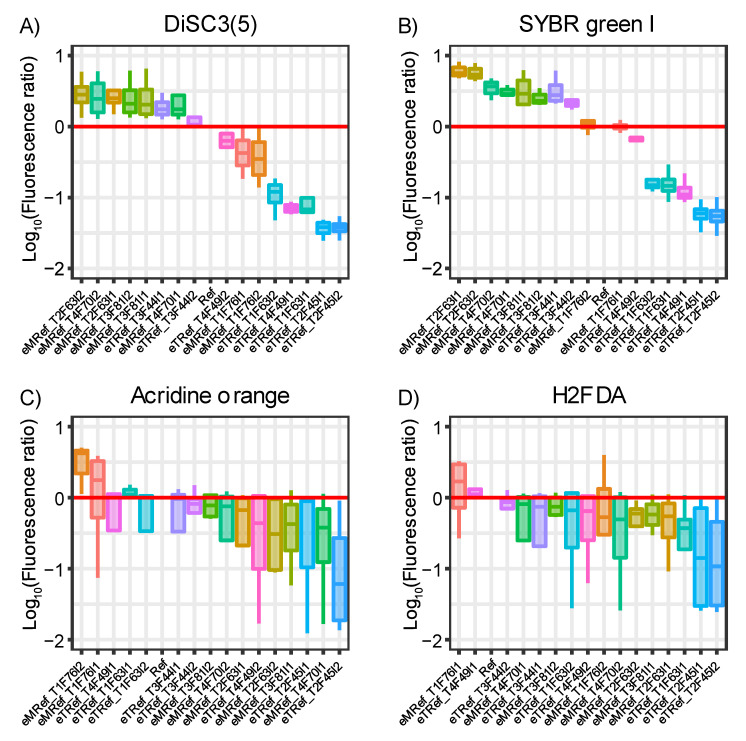
Intracellular levels of fluorophores in the evolved strains *eTRef* and *eMRef* in comparison to the parental strain ‘*Ref*’. The accumulation of these 4 fluorophores was depicted as the ratio of the median fluorescence signals for 16 different clonal isolates (abscissa) against that of the ‘*Ref*’. The data was log-transformed. Thus, a unit difference in the ordinate corresponds to a ten-fold difference. The red line indicates no difference observed. The data distribution and median of at least four biological replicates are represented in each box plot. (**A**) DiSC3(5), (**B**) SYBR green I, (**C**) Acridine orange, and (**D**) H2FDA. Names of the clonal isolates were abbreviated based on each TALE lineage identifier, flask number from which evolved clones were derived, and the isolate number (TALE 1 to TALE 4: T1 to T4, Flask number: F#, and Isolate 1 and Isolate 2: I1 and I2).

The observed fluorophore signal ratios of most fluorophores suggest a differential accumulation of several fluorophores among the evolved mutants. The fluorophore accumulation profiles of the tested clones against the full list of fluorophores are provided in Supplementary Figure S4 (separate file). Among those profiles, DiSC3(5), SYBR Green I, Acridine orange, and H2FDA were the most contrastive among *eTRef* and *eMRef* evolved mutants (Figure 7). The fluorescence signals of DiSC3(5) indicate that most of the *eTRef* evolved mutants showed approximately ten-fold less accumulation of DiSC3(5) than the ancestral strain ‘*Ref*’. This trend was also observed with SYBR green I. Both clones *eTRef*_T3F63I1/2 represented an exception by showing higher accumulation of both fluorophores relative to ‘*Ref*’. On the other hand, most of the *eMRef* evolved mutants showed a higher accumulation of both fluorophores relative to ‘*Ref*’. However, both clones derived from the hypermutator strain (*eMRef*_T1F76I1/2) showed a higher or a similar accumulation of DiSC3(5) and SYBR green I, respectively, relative to ‘*Ref*’. The intracellular levels of both H2FDA and acridine orange observed in most of clones derived from *eTRef* and *eMRef* evolved mutants were consistently below those of ‘*Ref*’, despite the presence of a few exceptions that showed higher accumulation of these fluorophores (Figure 7C,D). Given the canonical functions of MDTs and their promiscuity [6], those fluorophores that were differentially accumulated intracellularly among different mutants could be considered as potential ligands to estimate the changes in membrane transport of the evolved mutants.

**Figure 8:**
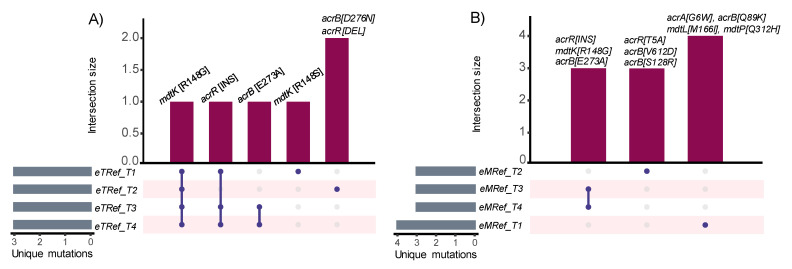
Overview of mutations associated with MDTs and their regulation in the evolved strains *eTRef* and *eMRef*. The UpSetR [110] plot depicts the possible convergence of mutations among all endpoint clonal isolates derived from (**A**) *eTRef* and (**B**) *eMRef* lineages. The *mdtK*(R148G) mutation occurred most frequently and was shared among independent parallel replicates in association with different mutations. The four parallel lineages are abbreviated to T1, T2, T3, and T4. Mutations from both clonal isolates were combined as one set and replicates were represented only once.

Converged mutations that occur in parallel evolution experiments provide evidence of a common adaptive trajectory between microbes exposed to the same conditions [37]. In addition, convergence may vary by mutational type [108]. Converged mutations associated with MDTs and their regulations among *eTRef* and *eMRef* evolved lineages are summarized in (Figure 8). Overall, the convergence is much higher on the level of genes than on mutational types or specific sites. In both *eTRef* and *eTRef* evolved mutants, mutations affecting *mdtK*, *acrB,* and *acrR* occurred most frequently among all isolates, but in different associations with other mutational types or genes. Specifically, the *acrR*(INS) mutation occurred once with *mdtK*(R148S) and once with *mdtK*(R148G) in *eTRef*_T1F63I1 and *eTRef*_T1F63I2, respectively. These clones showed a notebly low fluorophore accumulation compared to ‘*Ref*’. However, the co-occurrence of mutations in *acrR*(DEL), *mdtK*(R148G), and *acrB*(D276N) was correlated with the lowest values of fluorescence signals (as observed explecitly in *eTRef*_T2F45I1/2). A similar combination of mutations in *acrR*(INS), *mdtK*(R148G), and *acrB*(E273A) occurred frequently among mutants derived from both *eTRef* and *eMRef* that showed low fluorescence signals, but with some exceptions that showed high fluorescence signals relative to ‘*Ref*’. Evolved mutants derived from *eMRef* lineages generally showed contrastive fluorescence signals. These mutants harbor mutations that occurred less frequently and most of them were reported in clones derived from the hypermutator lineage A1 (i.e., *eMRef*_T1F76I1/2). Overall, these results mirrored the high frequency of adaptive convergence in the *eTRef* mutants and hinted at the potential of the *mdtK*(R148G) mutation in combination with *acrR*(DEL) and *acrB*(D276N) as an effective association that provided broader transport versatility. This is also in line with the acquired cross-cation resistance observed among *eTRef* evolved isolates (Section 3.2). Despite the emergence of other associations that showed similar or opposite outcomes, their broader effects or interactions are yet to be explored, possibly in terms of epistasis [109].

## 4. Conclusions

Functional redundancy and promiscuity of MDTs represent major challenges towards their identification and modulation in vivo. In this work, a multistep TALE approach was introduced to address these challenges. Briefly, the wild-type *E. coli* K-12-MG1655 and its cognate knockout individual mutants Δ*emrE*, Δ*tolC*, and Δ*acrB* under separate incremental supplementation of two lipophilic cations, TPP^+^ and MTPP^+^. During TALE experiments, the concentrations of both cations were tuned according to the actual rate of evolutionary adaptation. In that way, the evolved strains acquired a high level of cation resistance relative to the ancestral strains, allowing the selection of apparent as well as redundant MDTs and potential mutational mechanisms that enhance their actions.

Overall, three main outcomes of this study are outlined. First, the TALE approach effectively identified mutations which could be linked to nine MDTs. Specifically, three MDTs including *acrB*, *mdtK*, and *mdfA* were frequently mutated and commonly shared among all lineages evolved in both cations. In addition to these three MDTs, *acrE* was frequently mutated only in Δ*acrB* evolved mutants. The other MDTs including *emrD*, *tolC*, *acrA*, *mdtL*, and *mdtP* were less frequently mutated and explicitly found in MTPP^+^-evolved strains. This further emphasizes the flexibility of the *E. coli* multidrug effluxome that provides a very robust backup response under higher drug doses. However, the functional role of all these identified MDTs is consistent with previous studies (see Section 2.3), others such as *emrD*, *mdtL*, and *mdtP* are poorly characterized. Nevertheless, the experimental context used here can provide a guiding point to further evaluate their specific contribution to multidrug resistance. Second, diverse mutational mechanisms appear to modulate the activity of MDTs, more than 85% of them were shared among all strains evolved in both conditions (Figure 4). This highlights the overlapping specificity of these MDTs and confirms the observed cross-cation resistance (Figure 2 and Figure 3). This set of mutations serves as a promising pool of edits to both modulate MDTs and for use in identifying adaptive mutations in pathogenic strains. Third, the recognized structural mutations in the proximal binding domain of *acrB* and the permeation pathways of both *mdtK* and *mdfA* are consistent with previous investigations (see Section 3.3.2). Such insightful molecular details of multidrug recognition and transport can enhance our understanding of drug-protein interactions in vivo. Thus, the recognized mutational changes can reliably pair with other approaches such as molecular dynamic simulations to provide guiding principles for identifying drug permeation pathways, and consequently aid in the rational design of MDTs inhibitors.

## Figures and Tables

**Table 1:** Mutations that targeted unique ORFs or ORF intergenic regions identified for potential multidrug transporters (MDTs). Represented are the converged mutations that occurred in parallel evolution experiments (*n* = 4). The number of occurrences represents the number of parallel replicates at which a given mutation occurred. Exceptions that are represented with number of occurrences = 1 are explained in the table legend.

Strain	Cation	Gene	Mutation Types (Unique Counts)	Product	Number of Occurrences across Replicates (*n* = 4) ^a,b,c^
*eTRef*	TPP^+^	*ybjG/mdfA*	SNP (2)	undecaprenyl pyrophosphate phosphatase/multidrug efflux system protein	1 *
		*acrB*	SNP (4)	multidrug efflux system protein	4
		*mdtK*	SNP (3)	multidrug efflux system transporter	4
*eT*Δ*emrE*		*mdtK*	SNP (3)	multidrug efflux system transporter	3
		*acrB*	SNP (6)	multidrug efflux system protein	4
		*ybjG/mdfA*	MOB (1)	undecaprenyl pyrophosphate phosphatase/multidrug efflux system protein	2
			SNP (1)		1 ^a^
		*ribC/mdtK*	SNP (1)	riboflavin synthase, alpha subunit/multidrug efflux system transporter	1 ^c^
		*mdfA*	SNP (1)	multidrug efflux system protein	1 ^b^
*eT*Δ*tolC*		*mdtK*	SNP (2)	multidrug efflux system transporter	3
		*ribC/mdtK*	DEL (1)	riboflavin synthase, alpha subunit/multidrug efflux system transporter	2
			SNP (1)		2
*eT*Δ*acrB*		*ribC/mdtK*	SNP (1)	riboflavin synthase, alpha subunit/multidrug efflux system transporter	1 ^b^
		*mdtK*	SNP (4)	multidrug efflux system transporter	2
		*acrS/acrE*	MOB (3)	acrAB operon transcriptional repressor/cytoplasmic membrane lipoprotein	4
*eMRef*	MTPP^+^	*acrB*	SNP (8)	multidrug efflux system protein	4
		*acrA*	SNP (2)	multidrug efflux system protein	1 ^c^
		*ribC/mdtK*	SNP (1)	riboflavin synthase, alpha subunit/multidrug efflux system transporter	1 *^,b^
		*mdtK*	SNP (2)	multidrug efflux system transporter	3
		*mdtL*	SNP (1)	multidrug efflux system protein	1 *^,b^
		*mdtP*	SNP (1)	outer membrane factor of efflux pump	1 *^,b^
		*ybjG/mdfA*	SNP (1)	undecaprenyl pyrophosphate phosphatase/multidrug efflux system protein	1 ^b^
*eM*Δ*emrE*		*nudF/tolC*	SNP (1)	ADP-ribose pyrophosphatase/transport channel	1 ^c^
			DEL (1)		3
		*acrB*	SNP (7)	multidrug efflux system protein	4
		*mdtK*	SNP (1)	multidrug efflux system transporter	2
		*ybjG/mdfA*	SNP (2)	undecaprenyl pyrophosphate phosphatase/multidrug efflux system protein	2
*eM*Δ*tolC*		*ybjG/mdfA*	SNP (2)	undecaprenyl pyrophosphate phosphatase/multidrug efflux system protein	4
			INS (2)		4
		*mdfA*	SNP (2)	multidrug efflux system protein	1 ^a^
		*tisB/emrD*	SNP (1)	toxic membrane persister formation peptide, LexA-regulated/multidrug efflux system protein	1 ^b^
*eM*Δ*acrB*		*ybjG/mdfA*	SNP (1)	undecaprenyl pyrophosphate phosphatase/multidrug efflux system protein	3
			INS (2)		2
		*acrS/acrE*	MOB (3)	acrAB operon transcriptional repressor/cytoplasmic membrane lipoprotein	3
			SNP (2)		4

* Mutations were reported in one hypermutator population and/or a clonal isolate derived from it. ^a^ Mutations were reported in the intermediate point of only one TALE replicate (considered as an adaptive mutation in the early evolutionary trajectory). ^b^ Mutations were reported in the end point of only one TALE replicate (considered as an adaptive mutation in the late evolutionary trajectory). ^c^ Fixed mutation that was reported in at least one intermediate point and the end point samples derived from one TALE replicate. (Abbreviations: SNP: single-base substitution, MOB: mobile element insertion, DLE: deletion, and INS: insertion).

**Table 2:** Structural mutations targeted unique ORFs identified for potential multidrug transporters (MDTs). Depicted are the amino acid substitutions in MDTs reported for multiple strains evolved in both experimental conditions. Hydropathy indices are reported from [51].

MDTs	Strain	Mutations	Occurrence (*n* = 4)	Hydropathy Index/Polarity/Acidity Change
*acrB*	*eTRef*	D276G ^PD^ Asp → Gly	(2)	−3.5/Polar/Acidic → −0.4/Nonpolar/Neutral
		D276N ^PD^ Asp → Asn	(2)	−3.5/Polar/Acidic → −3.5/Polar/Neutral
		E273A ^PD^ Glu → Ala	(2)	−3.5/Polar/Acidic → 1.8/Nonpolar/Neutral
		N68K ^PD^ Asn → Lys	(1)	−3.5/Polar/Neutral → −3.5/Polar/Basic
	*eMRef*	V612D ^PD^ Val → Asp	(1)	4.2/Nonpolar/Neutral → −3.5/Polar/Acidic
		E273A ^PD^ Glu → Ala	(2)	−3.5/Polar/Acidic → 1.8/Nonpolar/Neutral
		D276G ^PD^ Asp → Gly	(2)	−3.5/Polar/Acidic → −0.4/Nonpolar/Neutral
		N274K ^PD^ Asn → Lys	(1)	−3.5/Polar/Neutral → −3.9/Polar/Basic
		V139F ^PD^ Val → Phe	(1)	4.2/Nonpolar/Neutral → −2.8/Nonpolar/Neutral
		Q125K ^PD^ Gln → Lys	(1)	−3.5/Polar/Neutral → −3.9/Polar/Basic
		S128R ^PD^ Ser → Arg	(1)	−0.8/Polar/Neutral → −4.5/Polar/Basic(strongly)
		Q89K ^PD^ Gln → Lys	(1)	−3.5/Polar/Neutral → −3.9/Polar/Basic
	*eT*Δ*emrE*	D276G ^PD^ Asp → Gly	(2)	−3.5/Polar/Acidic → −0.4/Nonpolar/Neutral
		N274K ^PD^ Asn → Lys	(1)	−3.5/Polar/Neutral → −3.9/Polar/Basic
		E273A ^PD^ Glu → Ala	(1)	−3.5/Polar/Acidic → 1.8/Nonpolar/Neutral
		E130V ^PD^ Glu → Val	(1)	−3.5/Polar/Acidic → 4.2/Nonpolar/Neutral
		Q125K ^PD^ Gln → Lys	(1)	−3.5/Polar/Neutral → −3.9/Polar/Basic
		N70K ^PD^ Asn → Lys	(1)	−3.5/Polar/Neutral → −3.9/Polar/Basic
	*eM*Δ*emrE*	V612D ^PD^ Val → Asp	(1)	4.2/Nonpolar/Neutral → −3.5/Polar/Acidic
		L542R ^TMD^ Leu → Arg	(1)	3.8/Nonpolar/Neutral → −4.5/Polar/Basic(strongly)
		D276G ^PD^ Asp → Gly	(1)	−3.5/Polar/Acidic → −0.4/Nonpolar/Neutral
		E273A ^PD^ Glu → Ala	(1)	−3.5/Polar/Acidic → 1.8/Nonpolar/Neutral
		V139F ^PD^ Val → Phe	(2)	4.2/Nonpolar/Neutral → −2.8/Nonpolar/Neutral
		Q89K ^PD^ Gln → Lys	(1)	−3.5/Polar/Neutral → −3.9/Polar/Basic
		V32A ^PD^ Val → Ala	(2)	4.2/Nonpolar/Neutral → 1.8/Nonpolar/Neutral
*mdtK*	*eTRef*	R148S Arg → Ser	(2)	−4.5/Polar/Basic(strongly) → −0.8/Polar/Neutral
		M1M Met → Met	(1)	1.9/Nonpolar/neutral → 1.9/Nonpolar/neutral (synonymous)
		R148G Arg → Gly	(4)	−4.5/Polar/Basic(strongly) → −0.4/Nonpolar/neutral
	*eMRef*	R81C Arg → Cys	(2)	−4.5/Polar/Basic(strongly) → 2.5/Polar/Neutral
		R148G Arg → Gly	(2)	−4.5/Polar/Basic(strongly) → −0.4/Nonpolar/neutral
	*eT*Δ*emrE*	R81C Arg → Cys	(1)	−4.5/Polar/Basic(strongly) → 2.5/Polar/Neutral
		R81L Arg → Leu	(1)	−4.5/Polar/Basic(strongly) → 3.8/Nonpolar/Neutral
		R148G Arg → Gly	(3)	−4.5/Polar/Basic(strongly) → −0.4/Nonpolar/neutral
	*eM*Δ*emrE*	R148G Arg → Gly	(2)	−4.5/Polar/Basic(strongly) → −0.4/Nonpolar/neutral
	*eT*Δ*tolC*	M1M ^b^ Met → Met	(1)	1.9/Nonpolar/Neutral → 1.9/Nonpolar/Neutral (No change)
		G428A Gly → Ala	(1)	−0.4/Nonpolar/Neutral → 1.8/Nonpolar/Neutral
	*eM*Δ*acrB*	W54G Trp → Gly	(1)	−0.9/Nonpolar/Neutral → −0.4/Nonpolar/Neutral
		A294V Ala → Val	(1)	1.8/Nonpolar/Neutral → 4.2/Nonpolar/Neutral
		G424S Gly → Ser	(1)	−0.4/Nonpolar/Neutral → −0.8/polar/Neutral
		G428S Gly → Ser	(1)	−0.4/Nonpolar/Neutral → −0.8/polar/Neutral
*mdfA*	*eT*Δ*emrE*	A144V Ala → Val	(1)	1.8/Nonpolar/Neutral → 4.2/Nonpolar/Neutral
	*eM*Δ*tolC*	F134C Phe → Cys	(1)	−2.8/Nonpolar/Neutral → 2.5/Polar/Neutral
		M146I Met → Ile	(1)	1.9/Nonpolar/neutral → 4.5/Nonpolar/neutral
*acrA*	*eMRef*	T177T Thr → Thr	(1)	−0.7/Polar/neutral → −0.7/Polar/neutral (synonymous)
		G6W (1) Gly→ Trp	(1)	−0.4/Nonpolar/Neutral → −0.9/Nonpolar/neutral
*mdtL*	*eMRef*	M166I Met → Ile	(1)	1.9/Nonpolar/neutral → 4.5/Nonpolar/neutral
*mdtP*	*eMRef*	Q312H Gln → His	(1)	−3.5/Polar/Neutral → −3.2/Polar/Basic (Weakly)

^PD^ Porter Domain of *acrB*. ^TMD^ Trans-Membrane Domain of *acrB*.

## Data Availability

Raw sequence data reported in this paper have been deposited in the European Nucleotide Archive under Bioproject number PRJNA890058. The dataset supporting the results of this article is included in the article (and its Supplementary Files).

## Supplementary Materials

The following supporting information can be downloaded at: https://www.mdpi.com/article/10.3390/membranes12121264/s1, The dataset supporting the results of this article is included in the article (and its Supplementary files). Two files were attached separately: Supplementary File containing all supplementary figures, tables, and legends of the Supplementary File(s)/Data. Supplementary Data S1 File: Spreadsheet containing all the whole genome sequencing results. Figure S1: The full fitness trajectories of strains evolved independently under increasing concentrations of TPP^+^. Figure S2: The full fitness trajectories of strains evolved independently under increasing concentrations of MTPP^+^. Figure S3: A schematic of the general logic used for filtering potential mutations in response to cation stress. Figure S4: Intracellular levels of 46 fluorophores in *eMRef* and *eTRef* evolved isolates compared to the parental strain ‘*Ref*’. Table S1: Properties of the TALE experiments. Table S2: List of flasks from which genomic DNA samples were deposited for whole genome resequencing. Table S3: Mutations affecting transcriptional regulators of MDTs. Table S4: Plasmids and oligonucleotides used for generating knockout strains.
