# Supplementary material for: Understanding Functional Redundancy and Promiscuity of Multidrug Transporters in E. coli under Lipophilic Cation Stress
Source: Membranes (Basel). 2022 Dec 14;12(12):1264. doi: 10.3390/membranes12121264 (PMC9783932; doi:10.3390/membranes12121264)
Supplement: Supplementary file 1 [file membranes-12-01264-s001.zip › membranes-2038631_Supplementary Figure S4.pdf]

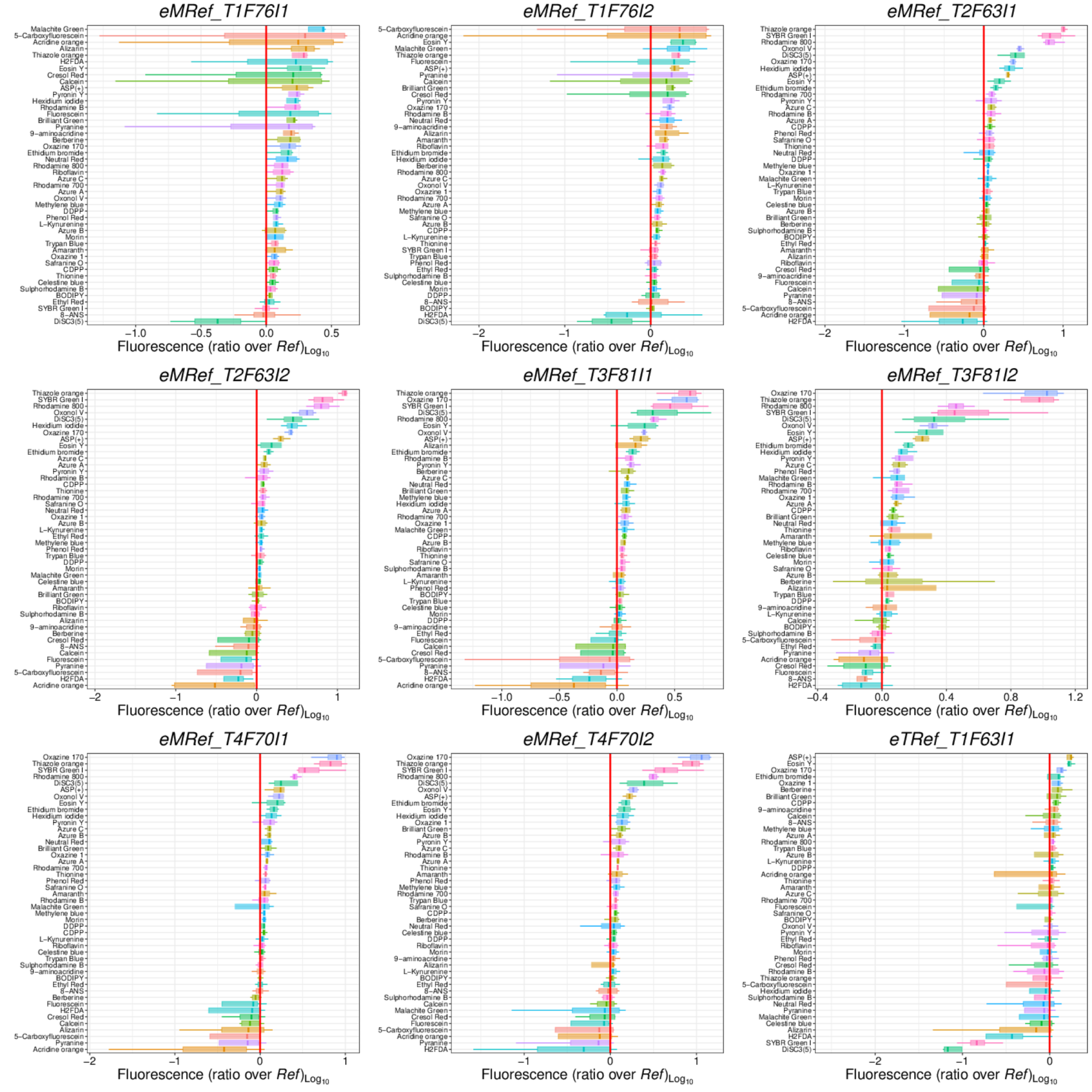
eMRef_T1F76I1
Malachite Green
5-Carboxyfluorescein
Acridine orange
Alizarin
Thiazole orange
H2FDA
Eosin Y
Cresol Red
Calcein
ASP(+)
Pyronin Y
Hexidium iodide
Rhodamine B
Fluorescein
Brilliant Green
Pyranine
9-aminoacridine
Berberine
Oxazine 170
Ethidium bromide
Neutral Red
Rhodamine 800
Riboflavin
Azure C
Rhodamine 700
Azure A
Oxonol V
Methylene blue
DDPP
Phenol Red
L-Kynurenine
Azure B
Morin
Trypan Blue
Amaranth
Oxazine 1
Safranine O
CDPP
Thionine
Celestine blue
Sulphorhodamine B
BODIPY
Ethyl Red
SYBR Green I
8-ANS
DiSC3(5)
−1.0
−0.5
0.0
0.5
Fluorescence (ratio over Ref)Log10
eMRef_T1F76I2
5-Carboxyfluorescein
Acridine orange
Eosin Y
Malachite Green
Thiazole orange
Fluorescein
ASP(+)
Pyranine
Calcein
Brilliant Green
Cresol Red
Pyronin Y
Oxazine 170
Rhodamine B
Neutral Red
9-aminoacridine
Alizarin
Amaranth
Riboflavin
Ethidium bromide
Hexidium iodide
Berberine
Rhodamine 800
Azure C
Oxonol V
Oxazine 1
Rhodamine 700
Azure A
Methylene blue
Safranine O
Azure B
CDPP
L-Kynurenine
Thionine
SYBR Green I
Trypan Blue
Phenol Red
Ethyl Red
Sulphorhodamine B
Celestine blue
Morin
DDPP
8-ANS
BODIPY
H2FDA
DiSC3(5)
−2
−1
0
Fluorescence (ratio over Ref)Log10
eMRef_T2F63I1
Thiazole orange
SYBR Green I
Rhodamine 800
Oxonol V
DiSC3(5)
Oxazine 170
Hexidium iodide
ASP(+)
Eosin Y
Ethidium bromide
Rhodamine 700
Pyronin Y
Azure C
Rhodamine B
Azure A
CDPP
Phenol Red
Safranine O
Thionine
Neutral Red
DDPP
Methylene blue
Oxazine 1
Malachite Green
L-Kynurenine
Trypan Blue
Morin
Celestine blue
Azure B
Brilliant Green
Berberine
Sulphorhodamine B
BODIPY
Ethyl Red
Amaranth
Alizarin
Riboflavin
Cresol Red
9-aminoacridine
Fluorescein
Calcein
Pyranine
8-ANS
5-Carboxyfluorescein
Acridine orange
H2FDA
−2
−1
0
1
Fluorescence (ratio over Ref)Log10
eMRef_T2F63I2
Thiazole orange
SYBR Green I
Rhodamine 800
Oxonol V
DiSC3(5)
Hexidium iodide
Oxazine 170
ASP(+)
Eosin Y
Ethidium bromide
Azure C
Azure A
Pyronin Y
Rhodamine B
CDPP
Thionine
Rhodamine 700
Safranine O
Neutral Red
Oxazine 1
Azure B
L-Kynurenine
Ethyl Red
Methylene blue
Phenol Red
Trypan Blue
DDPP
Morin
Malachite Green
Celestine blue
Amaranth
Brilliant Green
BODIPY
Riboflavin
Sulphorhodamine B
Alizarin
9-aminoacridine
Berberine
Cresol Red
8-ANS
Calcein
Fluorescein
Pyranine
5-Carboxyfluorescein
H2FDA
Acridine orange
−2
−1
0
1
Fluorescence (ratio over Ref)Log10
eMRef_T3F81I1
Thiazole orange
Oxazine 170
SYBR Green I
DiSC3(5)
Rhodamine 800
Eosin Y
Oxonol V
ASP(+)
Alizarin
Ethidium bromide
Rhodamine B
Pyronin Y
Berberine
Azure C
Neutral Red
Brilliant Green
Methylene blue
Hexidium iodide
Azure A
Rhodamine 700
Oxazine 1
Malachite Green
CDPP
Azure B
Riboflavin
Thionine
Safranine O
Sulphorhodamine B
Amaranth
L-Kynurenine
Phenol Red
BODIPY
Trypan Blue
Celestine blue
Morin
DDPP
9-aminoacridine
Ethyl Red
Fluorescein
Calcein
Cresol Red
5-Carboxyfluorescein
Pyranine
8-ANS
H2FDA
Acridine orange
−1.0
−0.5
0.0
0.5
Fluorescence (ratio over Ref)Log10
eMRef_T3F81I2
Oxazine 170
Thiazole orange
Rhodamine 800
SYBR Green I
DiSC3(5)
Oxonol V
Eosin Y
ASP(+)
Ethidium bromide
Hexidium iodide
Pyronin Y
Azure C
Phenol Red
Malachite Green
Rhodamine B
Rhodamine 700
Oxazine 1
Azure A
CDPP
Brilliant Green
Neutral Red
Thionine
Amaranth
Methylene blue
Riboflavin
Celestine blue
Morin
Safranine O
Azure B
Berberine
Alizarin
Trypan Blue
DDPP
9-aminoacridine
L-Kynurenine
Calcein
BODIPY
Sulphorhodamine B
5-Carboxyfluorescein
Ethyl Red
Pyranine
Acridine orange
Cresol Red
Fluorescein
8-ANS
H2FDA
−0.4
0.0
0.4
0.8
1.2
Fluorescence (ratio over Ref)Log10
eMRef_T4F70I1
Oxazine 170
Thiazole orange
SYBR Green I
Rhodamine 800
DiSC3(5)
ASP(+)
Oxonol V
Eosin Y
Ethidium bromide
Hexidium iodide
Pyronin Y
Azure C
Azure B
Neutral Red
Brilliant Green
Oxazine 1
Azure A
Rhodamine 700
Thionine
Phenol Red
Safranine O
Amaranth
Rhodamine B
Malachite Green
Methylene blue
Morin
DDPP
CDPP
L-Kynurenine
Riboflavin
Celestine blue
Trypan Blue
Sulphorhodamine B
9-aminoacridine
BODIPY
Ethyl Red
8-ANS
Berberine
Fluorescein
H2FDA
Cresol Red
Calcein
Alizarin
5-Carboxyfluorescein
Pyranine
Acridine orange
−2
−1
0
1
Fluorescence (ratio over Ref)Log10
eMRef_T4F70I2
Oxazine 170
Thiazole orange
SYBR Green I
Rhodamine 800
DiSC3(5)
Oxonol V
ASP(+)
Ethidium bromide
Eosin Y
Hexidium iodide
Oxazine 1
Brilliant Green
Azure B
Pyronin Y
Azure C
Rhodamine B
Azure A
Thionine
Amaranth
Phenol Red
Methylene blue
Rhodamine 700
Trypan Blue
Safranine O
CDPP
Berberine
Neutral Red
Celestine blue
DDPP
Riboflavin
Morin
9-aminoacridine
Alizarin
L-Kynurenine
BODIPY
Ethyl Red
8-ANS
Sulphorhodamine B
Calcein
Malachite Green
Cresol Red
Fluorescein
5-Carboxyfluorescein
Acridine orange
Pyranine
H2FDA
−1
0
1
Fluorescence (ratio over Ref)Log10
eTRef_T1F63I1
ASP(+)
Eosin Y
Oxazine 170
Ethidium bromide
Oxazine 1
Berberine
Brilliant Green
CDPP
9-aminoacridine
Calcein
8-ANS
Methylene blue
Azure A
Rhodamine 800
Trypan Blue
Azure B
L-Kynurenine
DDPP
Acridine orange
Thionine
Amaranth
Azure C
Rhodamine 700
Fluorescein
Safranine O
BODIPY
Oxonol V
Pyronin Y
Ethyl Red
Riboflavin
Morin
Phenol Red
Cresol Red
Rhodamine B
Thiazole orange
5-Carboxyfluorescein
Hexidium iodide
Sulphorhodamine B
Neutral Red
Pyranine
Malachite Green
Celestine blue
Alizarin
H2FDA
SYBR Green I
DiSC3(5)
−2
−1
0
Fluorescence (ratio over Ref)Log10

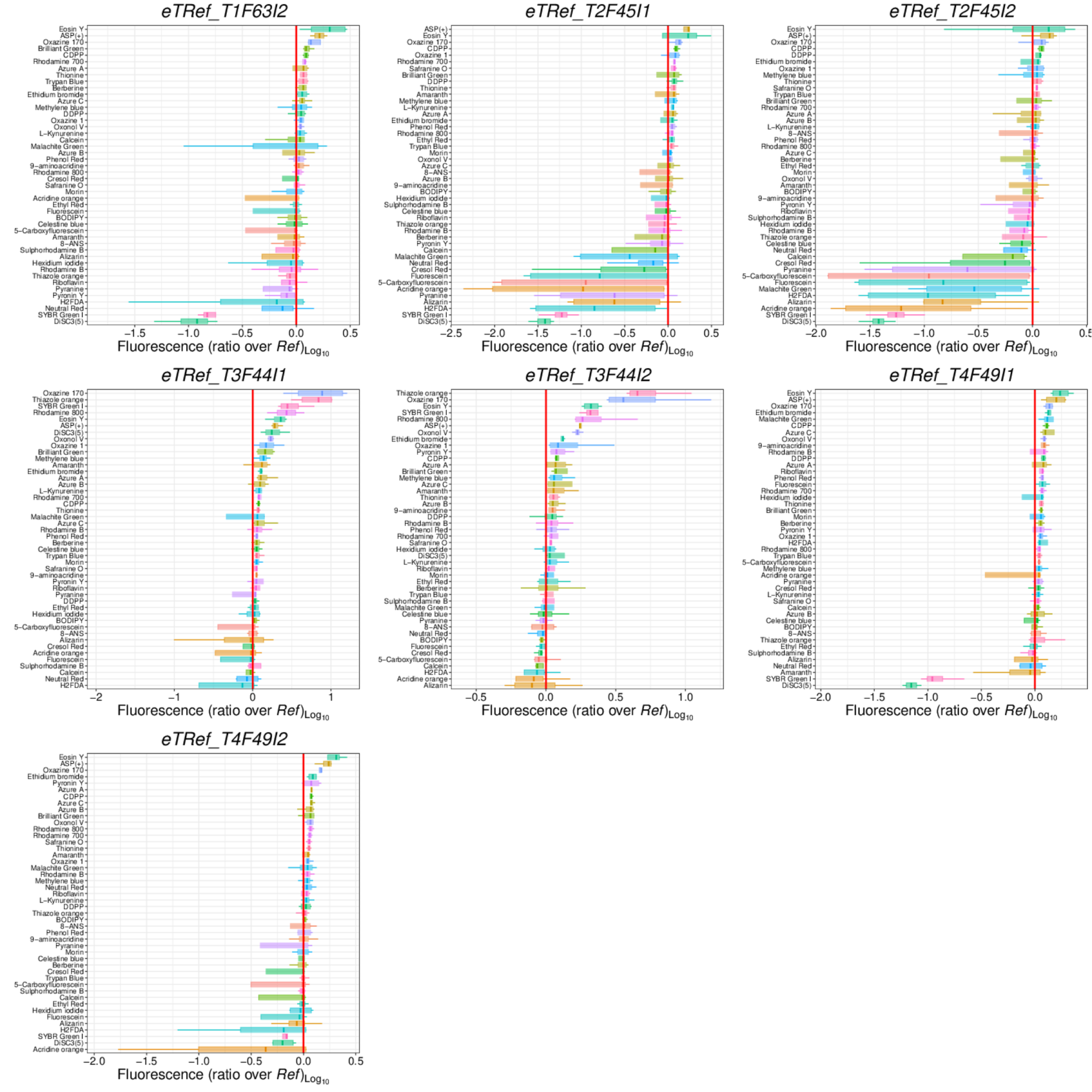

eTRef_T1F63I2
eTRef_T2F45I1
eTRef_T2F45I2
eTRef_T3F44I1
eTRef_T3F44I2
eTRef_T4F49I1
eTRef_T4F49I2
Fluorescence (ratio over Ref)Log10
