# Supplementary material for: Understanding Functional Redundancy and Promiscuity of Multidrug Transporters in E. coli under Lipophilic Cation Stress
Source: Membranes (Basel). 2022 Dec 14;12(12):1264. doi: 10.3390/membranes12121264 (PMC9783932; doi:10.3390/membranes12121264)
Supplement: Supplementary file 1 [file membranes-12-01264-s001.zip › membranes-2038631-Supplementary Materials.pdf]

membranes

Supplementary Material

Understanding Functional Redundancy and Promiscuity of Multidrug Transporters in *E. coli* under Lipophilic Cation Stress

Mohammad S. Radi [1], Lachlan J. Munro [1], Jesus E. Salcedo-Sora [2], Se Hyeuk Kim [1], Adam M. Feist [1,3,*] and Douglas B. Kell [1,4,*]

[1] Novo Nordisk Foundation Center for Biosustainability, Technical University of Denmark, Building 220, Kemitorvet, 2800 Kongens Lyngby, Denmark
[2] GeneMill, Shared Research Facilities, Faculty of Health and Life Sciences, University of Liverpool, Crown St., Liverpool L69 7ZB, UK
[3] Department of Bioengineering, University of California, 9500 Gilman Drive, La Jolla, San Diego, CA 92093, USA
[4] Department of Biochemistry and Systems Biology, Institute of Systems, Molecular and Integrative Biology, Faculty of Health and Life Sciences, University of Liverpool, Crown St., Liverpool L69 7ZB, UK
* Correspondence: afeist@ucsd.edu (A.M.F.); douglas.kell@liverpool.ac.uk (D.B.K.)

Supplementary Figures

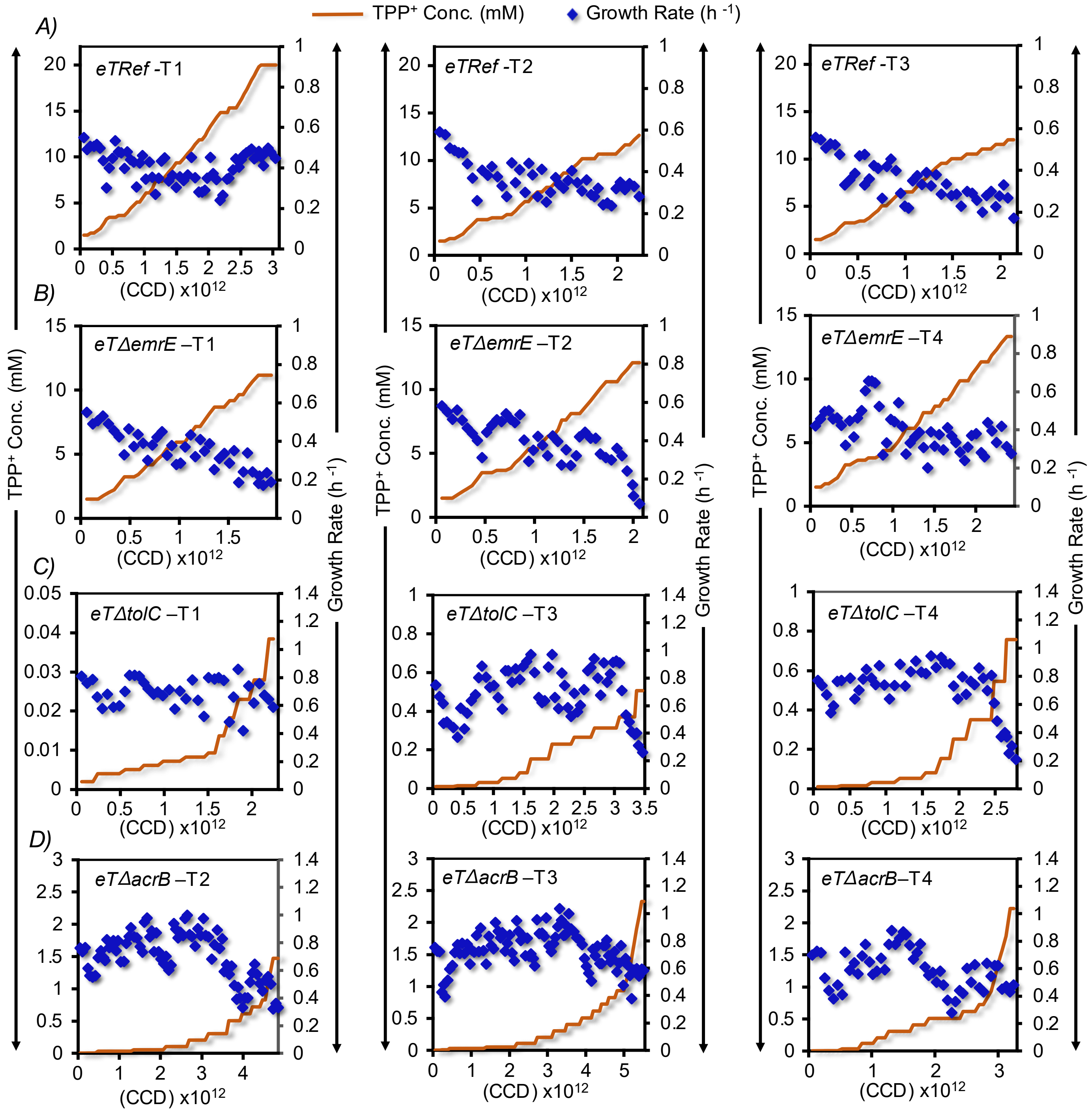

Figure S1. The full fitness trajectories of strains evolved independently under increasing concentrations of TPP^+. Plots represents the observed growth rate of three TALE replicate (n = 4 per each strain) that were growing under increasing concentrations of TPP^+. Each dot (bright blue diamonds) represents a calculated growth rate value of cells that were growing under increasing concentrations of TPP^+ (orange lines). Depicted are the full fitness trajectories and TPP^+ concentration (Conc., mM) versus cumulative cell divisions (CCDs) experienced by the cultures of (**A**) an evolved *E. coli* K-12-MG1655 (*eTRef*) TALE lineages (abbreviated to T) and its cognate knockout evolved lineages (**B**) *eTΔemrE*, (**C**) *eTΔtolC* and (**D**) *eTΔacrB*.

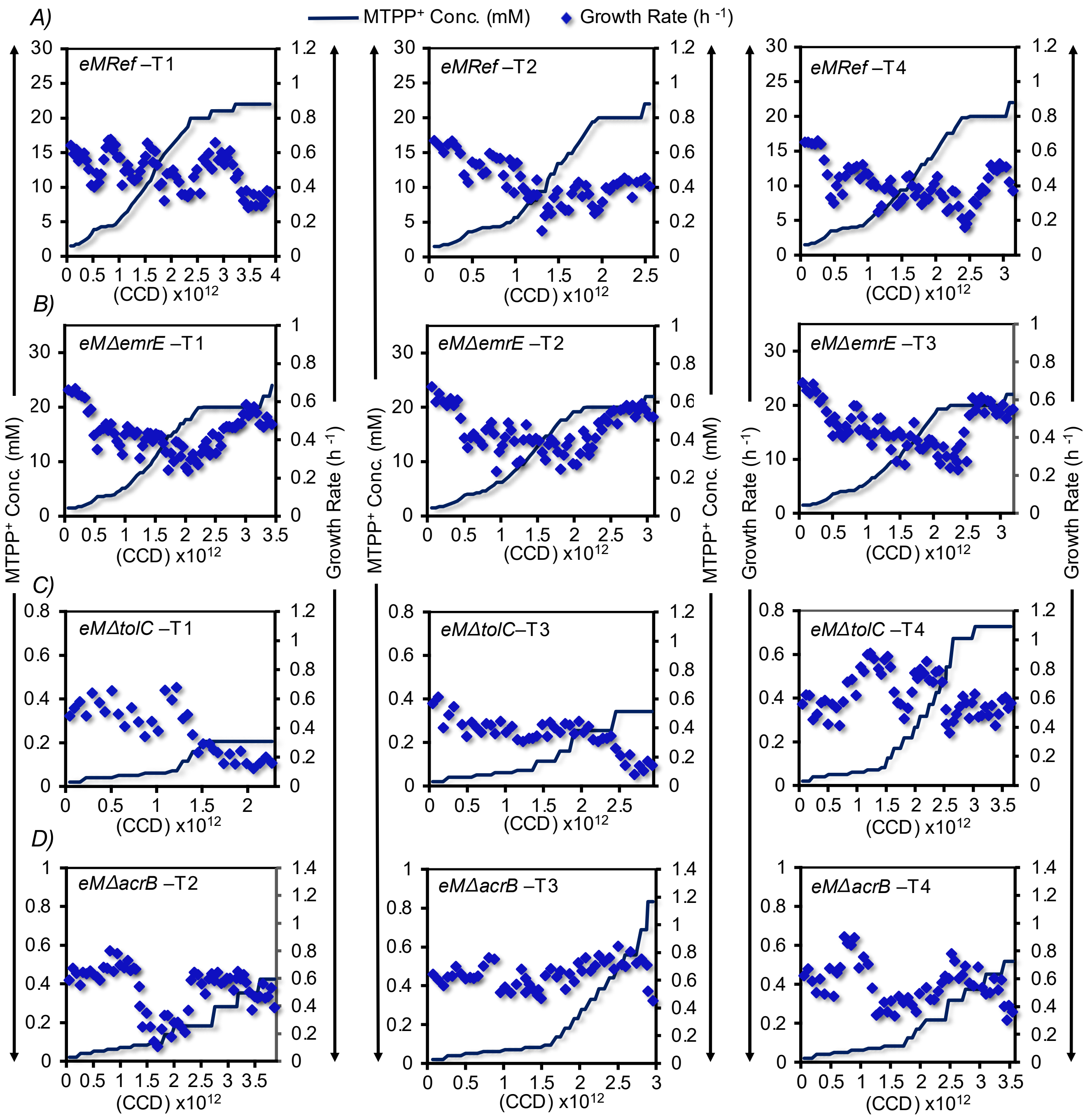

Figure S2. The full fitness trajectories of strains evolved independently under increasing concentrations of MTPP⁺. Plots represents the observed growth rate of three TALE replicate (n = 4 per each strain) that were growing under increasing concentrations of MTPP⁺. Each dot (bright blue diamonds) represents a calculated growth rate value of cells that were growing under increasing concentrations of MTPP⁺ (dark blue lines**)**. Depicted are the full fitness trajectories and MTPP⁺ concentration (Conc., mM) versus cumulative cell divisions (CCDs) experienced by the cultures of (**A**) an evolved *E. coli* K-12-MG1655 (*eMRef*) TALE lineages (abbreviated to T) and its cognate knockout evolved lineages (**B**) *eMΔemrE*, (**C**) *eMΔtolC* and (**D**) *eMΔacrB*.

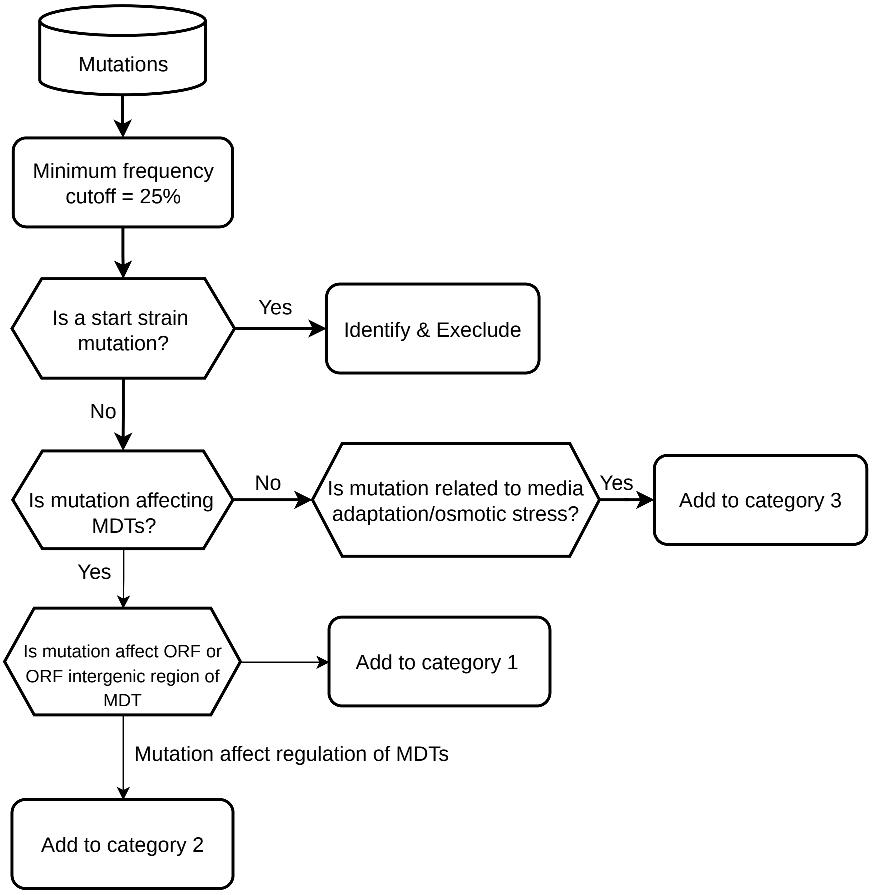

Figure S3. A schematic of the general logic used for filtering potential mutations in response to cation stress. Starting from the topmost box, mutations were categorized on a per condition basis on ALEdb database [1]. For population samples, the first step was to generate a frequency cutoff above which mutations will be evaluated. Given the distribution of all mutation frequencies and the typical standard deviation for calling a given mutation in a population, this baseline cut-off value was chosen to be at 25%. The next step downward is to identify and exclude start strain mutations by examining mutations shared by the starting strain clones and which were fully fixed in all the population samples and/or found in other evolution experiments in ALEdb database [1]. Details of the starting strain mutations are in Supplementary Data File S1. To generate a focus set of potential causal mutations in Multidrug Transporters (MDTs), mutations that were linked to MDTs functions were identified and listed if it occurs in samples of one or more TALE replicate. Further, mutations targeting a unique ORF or ORF intergenic region identified for MDTs listed as category 1, and mutations affecting the regulation of MDTs listed as category 2. Mutations that weren't associated with MDTs functions (i.e., general adaptation to growth in M9 minimal medium or general stress response) were also identified and categorized as category 3.

Figure S4 (Provided as a Separate File)

Legend for Supplementary Figure S4

Intracellular levels of 46 fluorophores in *eMRef* and *eTRef* evolved isolates compared to the parental strain *'Ref'*. The accumulation of these four fluorophores was depicted as the ratio of the median fluorescence signals for 16 different clonal isolates (abscissa) against that of the *'Ref'*. The data was log-transformed. Thus, a unit difference in the ordinate corresponds to a ten-fold difference. The red line indicates no difference observed. Each plot represents a different strain as indicated in each title. The data distribution and median of at least four biological replicates are represented in each box plot. The names of the fluorophores (abbreviated for some of them) are on the ordinate.

Table S1. Properties of the TALE experiments. Growth phenotypes of the endpoint evolved populations in each of the corresponding TALE experiments including the initial and the final concentrations of cations and the total number of cumulative cell divisions (CCD) experienced by the evolved cells.

Strain/Conditions	TALE#	Cation Concentration (mM)		Total CCD x10^{12}	*Avg. final growth rate (hr^{-1}) ± STDEV
		Initial	Final		
eTRef	**T1**	**1.5**	20	3.04	0.47 ± 0.02
	T2	1.5	12.65	2.24	0.31 ± 0.03
	T3	1.5	12	2.14	0.25 ± 0.08
	T4	1.5	16.52	2.74	0.30 ± 0.01
eTΔemrE	T1	1.5	11.16	1.93	0.19 ± 0.03
	T2	1.5	12.09	2.06	0.11 ± 0.05
	T3	1.5	14.27	2.25	0.20 ± 0.04
	T4	1.5	13.34	2.42	0.33 ± 0.07
eTΔtolC	T1	0.002	0.038	2.24	0.63 ± 0.04
	T2	0.002	0.757	2.58	0.16 ± 0.04
	T3	0.002	0.506	3.46	0.32 ± 0.07
	T4	0.002	0.757	2.79	0.25 ± 0.04
eTΔacrB	T1	0.002	2.76	5.65	0.40 ± 0.03
	T2	0.002	1.47	4.84	0.33 ± 0.01
	T3	0.002	2.33	5.52	0.57 ± 0.01
	T4	0.002	2.22	3.24	0.46 ± 0.02
eMRef	T1	1.5	22	3.88	0.35 ± 0.03
	T2	1.5	22	2.54	0.43 ± 0.02
	T3	1.5	24	3.62	0.52 ± 0.01
	T4	1.5	22	3.14	0.43 ± 0.07
eMΔemrE	T1	1.5	24	3.43	0.49 ± 0.02
	T2	1.5	22	3.07	0.53 ± 0.02
	T3	1.5	22	3.18	0.52 ± 0.02
	T4	1.5	22	2.91	0.54 ± 0.03
eMΔtolC	T1	0.02	0.205	2.26	0.17 ± 0.02
	T2	0.02	0.658	3.73	0.32 ± 0.08
	T3	0.02	0.342	2.92	0.13 ± 0.03
	T4	0.02	0.727	3.64	0.56 ± 0.03
eMΔacrB	T1	0.02	0.64	3.92	0.24 ± 0.01
	T2	0.02	0.42	3.86	0.46 ± 0.07
	T3	0.02	0.83	2.95	0.55 ± 0.05
	T4	0.02	0.51	3.55	0.35 ± 0.02

* Calculated from the last three flasks of each lineage.

Table S2. List of flasks from which genomic DNA samples were deposited for whole genome resequencing. Numbers from 1 to 4 represent the replicate number corresponding to each cation TALE experiment.

Strain/Conditions	TALE#	Intermediate points		Endpoints
		Point#1	Point#2	
eTRef	**1**	22	36	63
	2	20	33	45
	3	20	33	44
	4	20	34	49
eTΔemrE	1	20	35	40
	2	24	35	42
	3	20	34	48
	4	26	38	49
eTΔtolC	1	8	38	N/A*
	2	31	41	46
	3	42	56	62
	4	35	42	47
eTΔacrB	1	53	69	95
	2	47	63	81
	3	44	66	95
	4	19	36	53
eMRef	1	29	59	76
	2	28	40	63
	3	26	47	81
	4	29	52	70
eMΔemrE	1	27	48	74
	2	24	46	72
	3	29	51	74
	4	28	47	65
eMΔtolC	1	22	24	35
	2	23	35	64
	3	23	31	46
	4	35	46	63
eMΔacrB	1	N/A*	40	62
	2	N/A*	36	66
	3	N/A*	49	51
	4	N/A*	32	55

* These samples were omitted due to a contamination event confirmed during the TALE experiment. The subsequent flasks in Δ*acrB* TALE are evolved from previous clean points retrieved from the archived cryogenic stock.

Table S3. Mutations affecting transcriptional regulators of MDTs.

Strain	Gene	Mutation types (Unique counts)	Product	Number of occurrences across replicates (n=4)
eTRef	*acrR*	INS (2)	transcriptional repressor	4
		DEL (1)		1

eTΔemrE	*acrR*	INS (2)	transcriptional repressor	3
		SNP (1)		1
		DEL (1)		1
eMRef	*acrR*	SNP (4)	transcriptional repressor	4
		INS (2)		3
eMΔemrE	*acrR*	INS (5)	transcriptional repressor	3
		MOB (2)		2
		SNP (2)		1
	marR	SNP (2)	transcriptional repressor of multiple antibiotic resistance	2

Table S4. Plasmids and oligonucleotides used for generating knockout strains.

Plasmids	Genotype
pMP11	*araC Para::gam-bet-exo tetR PlacI::tetO::gRNA of pgRNA Pj23105::csn1 Am*^{R} *SC101(ts)*
pgRNA-bacteria	*Pj23119::gRNA Cm*^{R} *BR322*
pgRNA-emrE	*Pj23119::gRNA_emrE Cm*^{R} *BR322*
**Primers**	**Sequence**
dEmrE_MAGE	AAATATAAGAGCCTCCATATTTTAGTCGTTTAGAAACAAATTATTAGCATATTCTTTCCTGTTCAAACTGGAGAGAATTGTACTACAGTT
pgRNA_emrE_F	CGCTGGCTTATATTCCTACAGTTTTAGAGCTAGAAATAGCAAGTT
pgRNA_emrE_R	TTTCTAGCTCTAAAACTGTAGGAATATAAGCCAGCGACTAGTATT
dAcrB_FRT_F	CAGCCTGAACAGTCCAAGTCTTAACTTAAACAGGAGCCGTTAAGACATGCCTAATTTCTTTATCAATTAACCCTCACTAAAGGGCGG
dAcrB_FRT_R	GCATAAAAAAGGCCGCTTACGCGGCCTTAGTGATTACACGTTGTATCAATGATGATCGACAGTTAATACGACTCACTATAGGGCTC
dTolC_FRT_F	CAGTTTGATCGCGCTAAATACTGCTTCACCACAAGGAATGCAAATGAAGAAATTGCTCCCCAATTAACCCTCACTAAAGGGCGG
dTolC_FRT_R	CGTTGCCTTACGTTCAGACGGGGCCGAAGCCCCGTCGTCGTCATCAGTTACGGAAAGGGTTTAATACGACTCACTATAGGGCTC
emrE_Flank_F	ACCAGAGAAGAATGGGAAGG
emrE_Flank_R	ATGGTGACACCTGCTAACG
acrB_Flank_F	ATAACCAGCAAGCCGCAAGC
acrB_Flank_R	TACTCCTTAATGTTCGTAGG

Supplementary Note S1

The following fluorophores were used at 2 μM final concentration. SYBR Green I was present at a 104 fold dilution:

- ASP(+): 4-(4-(Dimethylamino)styryl-N-methylpyridinium (ASP+)
- CDPP: 3,6-Bis(4-chlorophenyl)-2,5-dihydropyrrolo[3,4-c]pyrrole-1,4-dione
- DDPP: 3,6-Diphenyl-2,5-dihydropyrrolo[3,4-c]pyrrole-1,4-dione
- BODIPY: Difluoro{2-[(3,5-dimethyl-2H-pyrrol-2-ylidene-N)methyl]-3,5-dimethyl-1H-pyrrolato-N}boron (BODIPY tetra-methyl)
- Hexidium iodide
- Morin
- Sulphorhodamine B
- 5-Carboxyfluorescein
- Acridine orange
- Alizarin
- Amaranth
- Brilliant Green
- Calcein
- Cresol Red
- Eosin Y
- Fluorescein
- H2FDA: 2',7'-dichlorodihydrofluorescein diacetate
- Malachite Green
- Neutral Red
- Pyronin Y
- Rhodamine B
- Riboflavin
- SYBR Green I
- Thiazole orange
- Rhodamine 800
- Azure A
- Azure B
- Azure C
- Celestine blue
- DiSC3(5)
- Ethyl Red
- L-Kynurenine
- Methylene blue
- Oxazine 1
- Oxazine 170
- Oxonol V
- Phenol Red
- Rhodamine 700
- Safranine O
- Thionine
- Trypan Blue
- 9-aminoacridine
- Berberine
- Pyranine
- Ethidium bromide

Supplementary Data S1:

Spreadsheet containing all the whole genome sequencing results.

Supplementary references

1. Phaneuf, P.V.; Gosting, D.; Palsson, B.O.; Feist, A.M. Aledb 1.0: A Database of Mutations from Adaptive Laboratory Evolution Experimentation. *Nucleic Acids Res.* **2019**, *47*, D1164–D1171, doi: 10.1093/nar/gky983.
